# Expression of chickpea CIPK25 enhances root growth and tolerance to dehydration and salt stress in transgenic tobacco

**DOI:** 10.3389/fpls.2015.00683

**Published:** 2015-09-08

**Authors:** Mukesh K. Meena, Sanjay Ghawana, Vikas Dwivedi, Ansuman Roy, Debasis Chattopadhyay

**Affiliations:** National Institute of Plant Genome ResearchNew Delhi, India

**Keywords:** *Cicer arietinum*, CIPK25, root, expression, salinity, dehydration

## Abstract

Calcium signaling plays an important role in adaptation and developmental processes in plants and animals. A class of calcium sensors, known as Calcineurin B-like (CBL) proteins sense specific temporal changes in cytosolic Ca^2+^ concentration and regulate activities of a group of ser/thr protein kinases called CBL-interacting protein kinases (CIPKs). Although a number of CIPKs have been shown to play crucial roles in the regulation of stress signaling, no study on the function of CIPK25 or its orthologs has been reported so far. In the present study, an ortholog of Arabidopsis CIPK25 was cloned from chickpea (*Cicer arietinum*). CaCIPK25 gene expression in chickpea increased upon salt, dehydration, and different hormonal treatments. CaCIPK25 gene showed differential tissue-specific expression. 5′-upstream activation sequence (5′-UAS) of the gene and its different truncated versions were fused to a reporter gene and studied in Arabidopsis to identify promoter regions directing its tissue-specific expression. Replacement of a conserved threonine residue with an aspartic acid at its catalytic site increased the kinase activity of CaCIPK25 by 2.5-fold. Transgenic tobacco plants overexpressing full-length and the high active versions of CaCIPK25 displayed a differential germination period and longer root length in comparison to the control plants. Expression of CaCIPK25 and its high active form differentially increased salt and water-deficit tolerance demonstrated by improved growth and reduced leaf chlorosis suggesting that the kinase activity of CaCIPK25 was required for these functions. Expressions of the abiotic stress marker genes were enhanced in the CaCIPK25-expressing tobacco plants. Our results suggested that CaCIPK25 functions in root development and abiotic stress tolerance.

## Introduction

Dehydration and salinity are the major abiotic stresses that account for the major loss of crop yield. As it is difficult to physically remove salt from the soil, improving crop tolerance to high salt becomes a critical task. Identification of molecular components conferring salt tolerance in plants can provide genetic markers or candidate genes for achieving this goal. Salinity causes injury to plant by ionic toxicity and osmotic stress that can also be induced by dehydration. These environmental stresses adversely affect photosynthesis, metabolism, and growth. Plants develop a variety of mechanisms to protect themselves from these environmental stresses. The major factors of plant responses to dehydration and salinity stress include perception and transduction of stress signals via signaling cascade and activation of stress-responsive genes. Calcium has been widely regarded as a ubiquitous second messenger of physiologically and environmentally induced signaling pathways in plants (Trewavas and Malhó, 1998). Internal and external stimuli such as developmental, light, and stresses elicit specific temporal changes in cytosolic Ca^2+^ concentration [Ca^2+^]_cyt_. The kinetics and magnitude of calcium ion concentration, i.e., “calcium signature” or [Ca^2+^]_cyt_ differs between different signals and possibly contributes to the specific response. These calcium signatures are decoded and transmitted by an array of Ca^2+^-binding proteins that relay this information into downstream responses. Calcineurin B-like (CBL) proteins are one of these protein families that functions by interacting with and activating CBL-interacting kinases (CIPKs) and, thereby, amplify and specify the signaling pathways.

CIPKs were first reported in Arabidopsis and grouped into sucrose non-fermented-1 (SNF-1) family kinases. SOS2 gene was identified in a genetic screening of salt overly sensitive (sos) mutants as a sucrose non-fermenting 1-like enzyme and subsequently, referred to as CIPK24 within the CIPK family (Liu et al., 2000; Kolukisaoglu et al., 2004). Genome-wide analysis has identified 26 CIPKs in Arabidopsis (Kolukisaoglu et al., 2004; Lyzenga et al., 2013), 33 CIPKs in rice (Kolukisaoglu et al., 2004; Piao et al., 2010), 27 CIPKs in poplar (Yu et al., 2007), and 43 CIPKs in maize (Chen et al., 2011). Extensive research in this area in the past few years led to the functional characterization of many CIPK genes from Arabidopsis and several other species and revealed their potential roles in abiotic stress tolerance. AtCIPK24/SOS2 was shown to be activated by interacting with AtCBL4/SOS3 and provided tolerance against salinity by phosphorylating and activating plasma membrane located Na^+^/H^+^ antiporter/SOS1 and, thereby, enhancing salt detoxification through Na^+^-extrusion into extracellular space (Liu and Zhu, 1997, 1998; Qiu et al., 2002). AtCIPK24/AtSOS2 was also found to interact with nucleoside triphosphate kinase 2 (NDPK2) as well as AtCAT2/AtCAT3, which were involved in reactive oxygen species (ROS) signaling and scavenging (Verslues et al., 2007) suggesting that AtCIPK24/AtSOS2 was a crucial regulator in the salt stress signaling network and was able to mediate both Na^+^ homeostasis and the oxidative stress response. AtCIPK23 and AtCIPK6 were reported to have a crucial role in K^+^ homeostasis. AtCBL1/AtCBl9-AtCIPK23 complex can directly activate the plasma membrane localized potassium channel AtAKT1, enhancing K^+^ uptake under low-K^+^ conditions (Xu et al., 2006). AtCBL4-AtCIPK6 complex was shown to modulate the activity of the K^+^-channel AKT2 by relocating it from endoplasmic reticulum membrane to the plasma membrane. Apart from AtCIPK23, its closest homolog AtCIPK9 also interacted with AtCBL3 to regulate K^+^ homeostasis under low K^+^ conditions (Liu et al., 2013). Apart from abiotic stress, CIPKs were also reported to participate in signaling related to development. AtCIPK19 loss-of-function mutant was impaired in pollen tube growth and polarity (Zhou et al., 2015). AtCIPK3, −9, −23, and −26 were shown to function downstream to CBL2 and −3 in maintaining magnesium homeostasis (Tang et al., 2015).

CIPKs from other plant species also displayed similar function as their orthologs in Arabidopsis. CIPK6 genes of chickpea (*Cicer arientinum*) and *Brassica napus* were shown to be involved in plant response to abiotic stress and abscisic acid signaling (Tripathi et al., 2009; Chen et al., 2012). Overexpression or suppression of SOS2 ortholog of apple MdSOS2 enhanced or reduced, respectively, the salinity tolerance in transgenic apple callus (Hu et al., 2012). Rice CIPK31 was found to be involved in germination and seedling growth under abiotic stress conditions in rice (Piao et al., 2010). Heterologous expression of cotton CIPK6 (GhCIPK6) in Arabidopsis significantly enhanced tolerance to multiple abiotic stresses (He et al., 2013). Expression of CIPK21 of maize (ZmCIPK21) enhanced salt tolerance in Arabidopsis (Chen et al., 2014). Previously, we reported a screening for drought-induced expression tag sequences (ESTs) of chickpea (Boominathan et al., 2004) and identified a putative CIPK-encoding EST. Deduced amino acid sequence of the full-length clone showed significant homology with Arabidopsis CIPK25 and, therefore, was named as CaCIPK25 (*C. arietinum* CIPK25). So far, no report on the characterization of CIPK25 of any plant is available in the literature. Here, we report a study on CaCIPK25 and showed that its expression in tobacco enhanced tolerance to salt and water-deficit stress. Further, replacement of a conserved threonine residue with aspartic acid in the kinase domain increased autokinase activity of CaCIPK25 and subsequently, stress-tolerance of the transgenic plants. We explored the tissue-specific expression of CaCIPK25 by fusing its 5′-upstream activation sequence (5′UAS) with a reporter gene. Different deletion constructs of 5′UAS were used to delineate the sequences responsible for expression in specific tissues.

## Methods and materials

### Plant materials, growth conditions, and treatments

Seeds of chickpea (*Cicer arietinum* L.) cv. BGD72 were surface sterilized and imbibed in water for overnight. Pre-soaked seeds were germinated in pots containing the agropeat: vermiculite (3:1) and grown in the growth chamber at 25°C ± 2°C and 40% relative humidity for 6 days in 10-h light conditions. For tissue specific expression analysis, root, stem, matured leaves, and flower of 90-day-old chickpea plants were used. For different treatments, 6 days-old pot-grown seedlings were used. The 6 days-old seedlings in pots were exposed to 4°C for cold stress. For salt or dehydration (20% polyethylene glycol 8000 w/v) stress, the roots of the seedlings were dipped in 250 mM sodium chloride or PEG solutions, respectively, for specified periods. For abscisic acid (ABA, 100 μM), salicylic acid (SA, 15 μM), and methyl jasmonate (MeJA, 100 μM) treatments, the solutions were sprayed on leaves and the roots were dipped in these solutions as well for the specified periods. The control samples were treated with water similarly. For auxin 5 μM IAA (indole acetic acid) and cytokinin (5 μM BAP) treatments, roots of the seedlings were dipped into the auxin and cytokinin solutions. Total RNA from the whole seedlings was used for gene expression.

### Construct preparation, transgenic plant development, and staining

Full length coding sequence (CDSs) was cloned by 5′ and 3′-RACE (rapid amplification of cDNA ends) using the primers mentioned in Supplementary Table 1 following previously described method (Tripathi et al., 2009). CDS of full-length and point-mutated CaCIPK25 were cloned in binary vector pBI121 for overexpression in *Nicotiana tabacum* var. Xanthi (tobacco). 2.3 kb long 5′-upstream activation sequence (UAS) along with 5′ untranslated region (UTR) of CaCIPK25 was retrieved from the chickpea genome sequence (Jain et al., 2013). Full-length and truncated 5′UAS along with the 5′UTR of CaCIPK25 were amplified by PCR using the primers mentioned in the Supplementary Table 1 and were cloned in pBI101 to drive expression of β-glucuronidase (GUS) and introduced in *Arabidopsis*. *In silico* analysis of the 5′-UAS was done using the tool PLACE (Higo et al., 1999). Transgenic *Arabidopsis* lines were made by floral dip method as described before (Tripathi et al., 2009). T_0_ seeds were screened for kanamycin (50 mg/l) resistance to identify independent transgenic lines. T_3_/T_4_ homozygous transgenic seeds were used for experiments. The *Agrobacterium*-mediated transformation of tobacco leaf explants was performed with *A. tumifaciens* gv 3101 as described earlier (Gelvin and Schilperoort, 1994). For salt tolerance experiments, seedlings grown on ½-strength Murashige-Skoog (MS) medium with 1.5% sucrose for 8 days were transferred to the same medium with or without 250 mM sodium chloride and kept for 10 days and returned to growth medium for recovery. Histochemical GUS staining was done by vacuum infiltration of GUS-staining solution composed of 50 mM sodium phosphate buffer pH 7.0, 2 mM EDTA, 0.12% Triton X-100, 0.4 mM potassium ferrocyanide, 0.4 mM potassium ferricyanide, 1.0 mM 5-bromo-4-chloro-3-indoxyl-beta-D-glucuronide cyclohexyl ammonium salt (X-Gluc) (Sigma-Aldrich, MO, USA) for 5–15 min and then incubated at dark from 2 to 12 h depending on tissue type. The stained tissues were cleared from chlorophyll by incubating in 70% ethanol at 65°C for 1 h and visualized by stereomicroscope.

### RNA isolation and expression analysis

Total RNA was extracted from different tissue samples using Trizol (Invitrogen, CA, USA). Eight-day-old seedlings of tobacco were used for RNA isolation. First strand cDNA was synthesized by High Fidelity cDNA synthesis kit (Roche Diagnostics GmbH, Germany) and oligo dT primer at 50°C for 30 min. Quantitative real-time PCR (qRT-PCR) experiments and calculations were performed using three technical and three biological replicates following the methods described before (Meena et al., 2015). Briefly, the reaction was performed in 10 μl reaction volume with 225 nM of each of the forward and reverse primers (Supplementary Table 2) and 2X Power SYBR Green PCR master mix (Applied Biosystems, CA, USA) using Vii A 7 Real-Time PCR System (Applied Biosystems, CA, USA). Chickpea Elongation factor 1-α (EF-1α) (GenBank: AJ004960.1) and tobacco actin (GenBank: BAD27408) genes were used as internal controls. Calculations were done using delta-delta Ct method. Paired students *t*-test was conducted to determine statistical significance of the results.

### Site-directed mutagenesis, bacterial expression, and kinase assay

Site-directed mutagenesis was done by replacing threonine (T) with aspartic acid (D) at 171^th^ position in CaCIPK25. Mutagenesis reactions were carried out on double-stranded plasmid DNA using Pfu Turbo DNA polymerase (Stratagene, La Jolla, CA) following parameters: 95°C for 30 s; 16 cycles of 95°C for 30 s, 58°C for 1 min, and 72°C for 7 min using primers CaCIPK25T/D171F and CaCIPK25T/D171R. The bacteria-derived template (double stranded plasmid DNA used as a template for PCR reaction) was digested with methylation-specific restriction enzyme Dpn I at 37°C for 6 h and the digested PCR product was transformed into *Escherichia coli* DH5α cells. The mutation and the fidelity of the rest of the construct were confirmed by DNA sequencing. For the bacterial expression, coding sequence of CaCIPK25 was cloned into pGEX4T-2 to produce glutathione-S-transferase (GST)-fused protein. The construct was introduced into *E. coli* BL21 (DE3)-codon plus. Protein expression was induced by 0.5 mM Isopropyl-β-D-thiogalactopyranoside (IPTG) for 3 h at 37°C. The bacterial pellet was suspended in lysis buffer (10 mM phosphate buffer pH7.0, 140 mM NaCl, 2.7 mM KCl, 0.5 mg lysozyme/gm of pellet) and was incubated for 1 h at 40°C. This cell suspension was sonicated 3 times with 20 s pulse and was centrifuged at 13,000 rpm at 4°C for 15 min to collect the supernatant. The GST-CaCIPK25 was affinity-purified using GSH-sepharose beads (GE Healthcare, USA). Kinase activity was measured as the incorporation of radioactivity from γ^32^P-ATP into the CaCIPK25 and myelin basic protein (MBP). The purified recombinant GST-CaCIPK25 protein (0.5 μg) and MBP (0.5 μg) were incubated in the kinase buffer (10 μCi γ^32^P-ATP, 20 mM Tris-HCl (pH 8.0), 5 mM MnCl_2_, 1 mM CaCl_2_, 0.1 mM EDTA, and 1 mM DTT) for 30 min at 30°C. The reaction was stopped by addition of 4 X SDS-sample buffer. Reaction samples were boiled for 5 min. to denature proteins and separated by 10% SDS-PAGE electrophoresis and viewed by autoradiography.

## Results

### Cloning and sequence analysis of CaCIPK25

**Figure 1:**
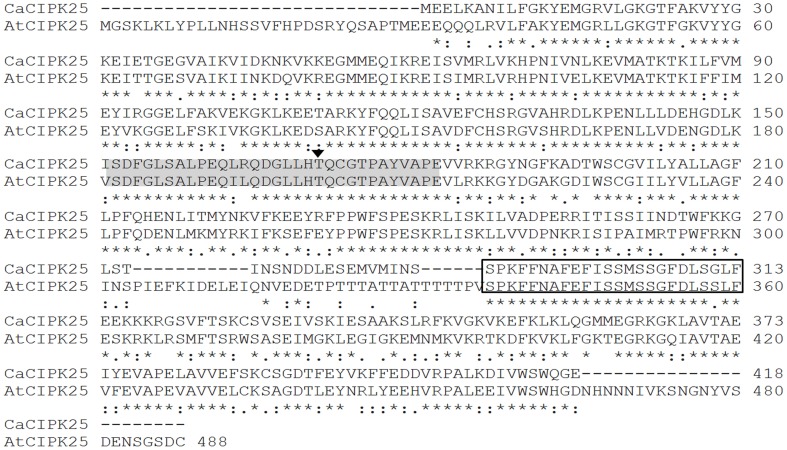
**Protein sequence alignment of AtCIPK25 and its ortholog CaCIPK25 in chickpea using ClustalW**. The activation loop is shown in shaded region with the conserved threonine residue marked with an arrow. The NAF/FISL module responsible for interaction with CBL is shown in box.

A previously reported screening to identify dehydration-inducible chickpea expression sequence tags (ESTs) (Boominathan et al., 2004) yielded an EST (GenBank: CD051323) with high expression under dehydration. 5′ and 3′ RACE (Rapid amplification of cDNA ends) resulted in a cDNA clone of 1727 base pair (bp) in length. Deduced protein sequence of the clone displayed highest homology (67% identity and 81% similarity) with CIPK25 of Arabidopsis and annotated as chickpea CIPK25 by NCBI (National Center for sBiotechnology Information), hence, referred to as CaCIPK25 (*C. arietinum* CIPK25, NCBI: XP_004498818). The protein sequence showed 88% identity with a *Medicago truncatula* CBL-interacting protein kinase (XP_003588823.1) and 74% identity with CIPK25 of *Theobroma cacao* (XP_007011728.1). In addition to the 1257 bp long protein-coding sequence (CDS), the cDNA clone also possessed a 5′-untranslated region (5′UTR) of 174 bases in length and a 3′-untranslated region (3′UTR) of 295 bases in length (Supplementary Text 1). Sequencing of the genomic DNA clone and comparison with the genome sequence showed that the gene was intronless and was located on linkage group 4. The deduced protein sequence was 418 amino acids in length with estimated molecular mass 47.55 kDa. CaCIPK25 was quite shorter in size than its *Arabidopsis* ortholog, which is of 488 aa, however, like other CIPKs, possessed an N-terminal SNF-1-related serine/threonine protein kinase domain (12–266 aa) and a C-terminal regulatory domain (295–409 aa) with a CBL-interacting NAF/FISL module. The activation loop (DFG…APE) and the threonine residue (Thr171), substitution of which by aspartic acid in SOS2 resulted in constitutive kinase activity, were conserved in CaCIPK25 (Figure 1).

### Stress-mediated and tissue-specific expression of CaCIPK25

**Figure 2:**
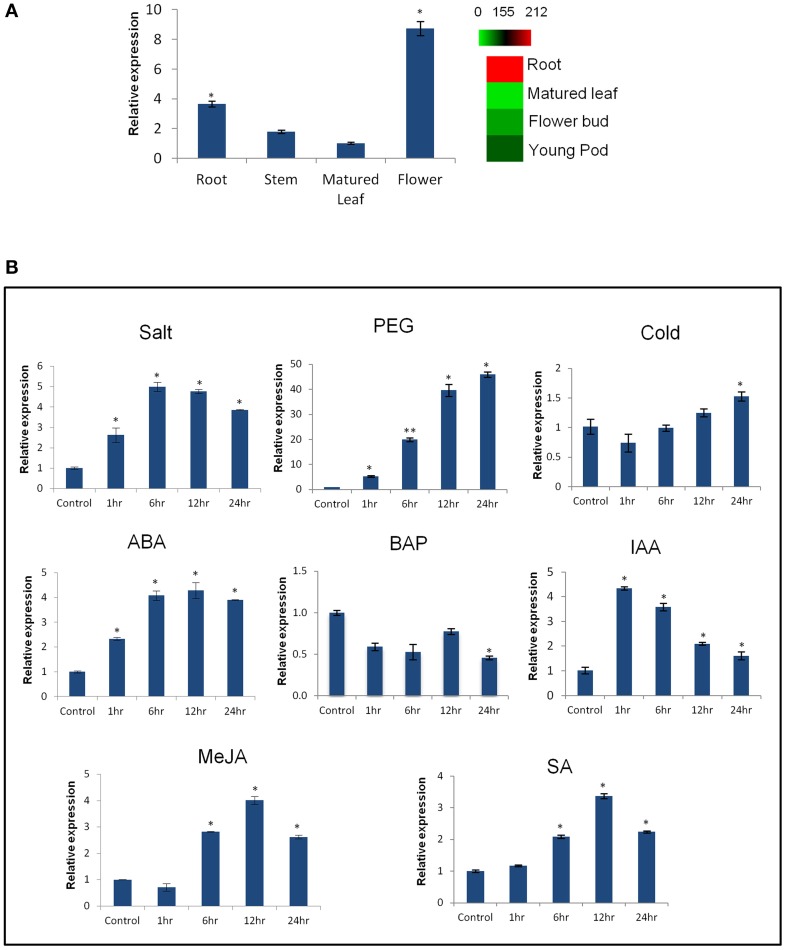
**Expression analysis of ***CaCIPK25*** gene in different tissues and under different treatments**. **(A)** Fold expression of CaCIPK25 gene, keeping its expression level in matured leaf as reference, in different tissues as assessed by qRT-PCR. Digital expression profile of the corresponding transcript using RNASeq data is shown by a heatmap. **(B)** Fold expression change of *CaCIPK25* gene in whole chickpea seedlings upon treatment with various stresses and hormones as mentioned. Chickpea *EF1*α gene was used as internal control. Values in the Y-axis describe fold-changes in expression. X-axis describes time periods. Standard deviations were derived from three biological replicates. ^*^, ^**^ indicate statistically significant difference (*p* < 0.05) and (*p* < 0.01), respectively, from the control sample.

**Figure 3:**
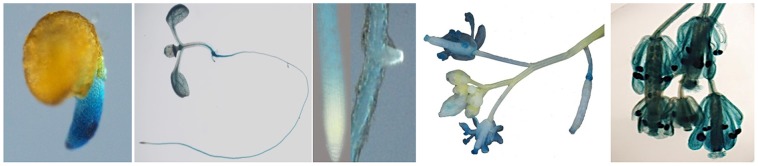
**Activity of ***CaCIPK25*** gene promoter in different tissues in transgenic ***Arabidopsis*****. CaCIPK25 gene promoter up to the translation start codon was fused to GUS gene and introduced in *Arabidopsis* to study promoter activity in different tissues. Representative samples were shown after histochemical GUS staining. Tissues shown from left were radicle, whole seedling, root apex, lateral root initial flower buds and flowers, flower at higher magnification.

Expression profiling of *CaCIPK25* by qRT-PCR in chickpea, showed its highest expression in flower was about 2.5-fold, while the expressions in stem and leaf were 0.5− and 0.3−folds, respectively, of its expression level in root. Similar tissue-specific expression profile of the corresponding transcript (TC05858) was reported using RNASeq data (Garg et al., 2011) (Figure 2A). Upon treatment with 250 mM of sodium chloride, *CaCIPK25* transcript level increased at 1 h by 2.8-fold, reached at 5-fold by the 6th h and then declined to 3.9-fold at the 24th h. *CaCIPK25* expression steadily increased by PEG treatment from 4-fold increase after 1 h to 48-fold increase after 24 h of treatment. Exposure to low-temperature did not affect its expression significantly. Both the ABA and auxin treatments enhanced *CaCIPK25* expression, however, with different time kinetics. The expression quickly increased to 4.4-fold within 1 h of IAA treatment and then slowly declined to 1.8-fold at 24th h, whereas, transcript level increased slowly to 4.1-fold after 12 h of treatment with ABA and decreased to 3.9-fold after 24th h. Auxin and cytokinin function antagonistically and synergistically in root development. Accordingly, BAP treatment reduced *CaCIPK25* expression by more than 2-fold after 6 and 24 h of treatment. Treatment with methyl jasmonate (MeJA) and salicylic acid (SA) resulted in similar expression profiles with a slow increase in transcript level to about 4-fold after 12 h and then decrease to about 2-fold after 24 h (Figure 2B). 2.2 kb upstream activation sequence (5′UAS/promoter) together with the 5′UTR (Supplementary Text 1) of CaCIPK25 gene was cloned and fused with the reporter gene β-glucuronidase (*GUS*) (pCaCIPK25-GUS) and introduced in Arabidopsis to monitor tissue-specific expression of the gene at different growth stages. pCaCIPK25*-GUS* displayed a strong expression all over the radicle just after germination. However, with growth, the primary and the lateral roots, except the root tips and lateral root initials comprising of meristem cells, showed strong expression of the gene. This differential GUS staining in root indicated that *CaCIPK25* promoter activity is suppressed in tissues with high auxin concentration. The cotyledons, mostly the veins, showed moderate GUS expression. However, the true leaves showed a low GUS staining. The stem did not show any detectable GUS expression. As expected, the strongest expression was observed in flower, specifically, in the petals, anthers, and stigma. The promoter activity in flower was dependent on growth stage as GUS activity was not visible in the immature flowers (Figure 3).

### Analysis of CaCIPK25 promoter for tissue-specific expression

**Figure 4:**
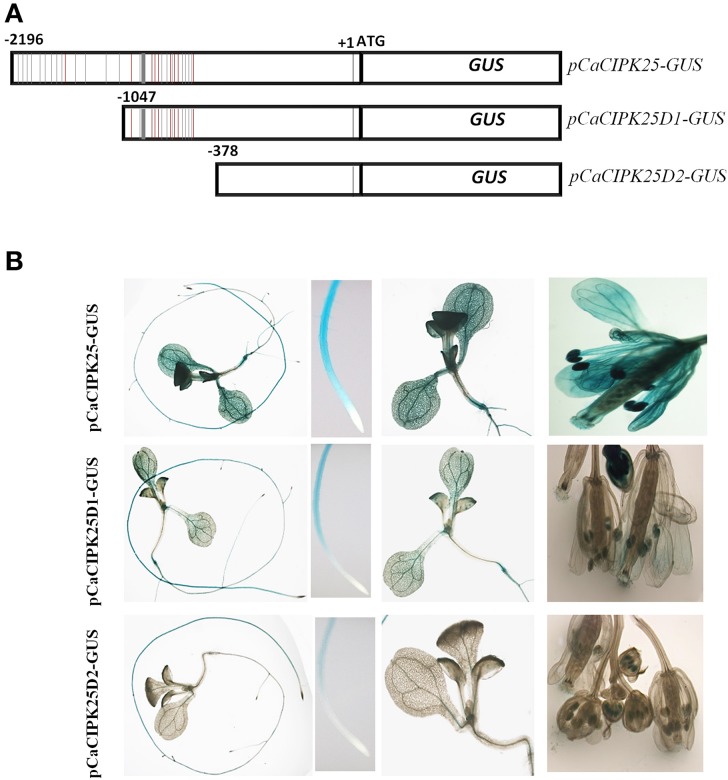
**Analysis of ***CaCIPK25*** promoter for tissue-specific expression**. **(A)** Schematic diagram of CaCIPK25 promoter-GUS reporter construct (*pCaCIPK25-GUS*) and its two truncated versions (*pCaCIPK25D1-GUS* and *pCaCIPK25D2-GUS*). Positions (not to scale) of ROOTMOTIFTAPOX1 and POLLEN1LELAT52 elements are shown in gray and red lines. Transcription and translation start sites (+1 and ATG) are marked. **(B)** Expression of GUS driven by different deletion constructs of *CaCIPK25* promoters in whole seedlings, root apex, cotyledon, and leaves and flowers are shown.

The 2.2 kb promoter sequence/5′UAS was analyzed for the presence of putative *cis*-acting elements. There were several dehydration responsive element/C-repeat (GTCGAC), ABA-responsive element (ACGTG), ARR1AT (NGATT), auxin responsive elements (TGTCTC), and W-box element (TTGAC/TGAC) are present within this region of the promoter explaining differential expression of CaCIPK25 upon treatments with salt, PEG, ABA, BAP, and IAA. In addition to those, multiple copies of ROOTMOTIFTAPOX1 (ATATT) (24 copies in the positive strand) and POLLEN1LELAT52 (AGAAA) (9 copies in the positive strand) elements, which drive root-specific and anther-specific expression, respectively, were found. Detail analysis of *cis*-acting elements in the 5′UAS of CaCIPK25 is presented in Supplementary Text. ROOTMOTIFTAPOX1 element was first identified in the promoter region of the rolD gene of *Agrobacterium rhizogenes*. The GUS gene driven by rolD promoter strongly expressed in the roots and expressed at a very low level in stem and leaves of tobacco plants. The distinctive expression pattern of the rolD promoter-GUS construct was that the strongest GUS activity was observed in the root elongation zone and vascular tissue and a very low expression in the root apex (Elmayan and Tepfer, 1994), highly consistent with the expression pattern of pCaCIPK25-GUS construct. A high GUS activity driven by CaCIPK25 promoter was observed in the root, cotyledon and reproductive organs and a very low GUS activity was observed in the true leaves. The pollen-specific *cis*-acting element POLLEN1LELAT52 was previously reported in the 5′UAS of tomato endo-β-mannase 5 gene (LeMAN5). Transgenic Arabidopsis plants expressing GUS driven by LeMAN5 promoter showed strong GUS activity in the anthers and pollens. In anthers, the highest LeMAN5 mRNA expression was observed in the later stages of flower development (Filichkin et al., 2004), similar to pCaCIPK25-GUS expression in flower. To delineate the *CaCIPK25* promoter regions driving the root and flower-specific expression, two promoter deletion constructs were used. The first deletion construct (pCaCIPK25D1) was made by retaining −1 to −1047 bases of the promoter. The first deletion of 1150 bases from the 5′-end removed 13 ROOTMOTIFTAPOX1 elements out of twenty-four and one POLLEN1LELAT52 elements out of nine (Figure 4A). Arabidopsis plants expressing GUS driven by pCaCIPK25D1 showed a substantial reduction of GUS-staining in cotyledons, petals, anthers, and stigma. However, decrease in GUS stain in roots was not so profound, suggesting the −1046 to −2196 region of the promoter was more relevant for the expression of the gene in cotyledon, petal and anther tissues. The second deletion construct (pCaCIPK25D2) removed next 700 bases and all the ROOTMOTIFTAPOX1 and POLLEN1LELAT52 elements. This deletion totally abolished GUS expression in petals and cotyledons. There was a substantial reduction in GUS expression in roots and anthers, but the removal of all the ROOTMOTIFTAPOX1 and POLLEN1LELAT52 elements did not totally abolish the GUS expression in these two tissues, suggesting that the immediate 378 bases from the transcription start site of the promoter was also responsible, although modestly, for root- and anther-specific expression (Figure 4B).

### Kinase activity of CaCIPK25

**Figure 5:**
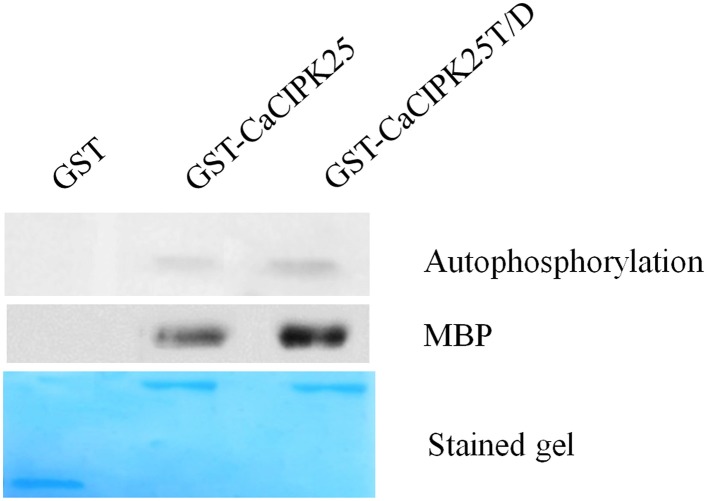
**Kinase assay of CaCIPK25 protein**. Autophosphorylation and substrate phosphorylation assay of bacterially expressed GST-CaCIPK25 and CaCIPK25T/D proteins. GST protein was used as control. Myelin basic protein (MBP) was used as substrate. A Coomassie blue-stained gel shows the amount of CaCIPK25 protein loaded.

In order to biochemically characterize the CaCIPK25 protein, the CDS was expressed in *E. coli* as a glutathione-S-transferase (GST)-fused protein. The conserved threonine (T171) residue located at the activation domain was substituted with aspartic acid (D) and mutated recombinant protein was expressed similarly. The purified recombinant proteins were tested for auto- and substrate phosphorylation. GST-CaCIPK25 showed a low level of auto-kinase activity. The T171D substitution increased the autokinase activity of the protein by about 2-fold. GST-CaCIPK25 was able to use myelin basic protein as a substrate and the aspartic acid substitution increased the kinase activity protein by about 2.5-fold using this substrate (Figure 5).

### Increased root length of CaCIPK25-expressing tobacco plants

**Figure 6:**
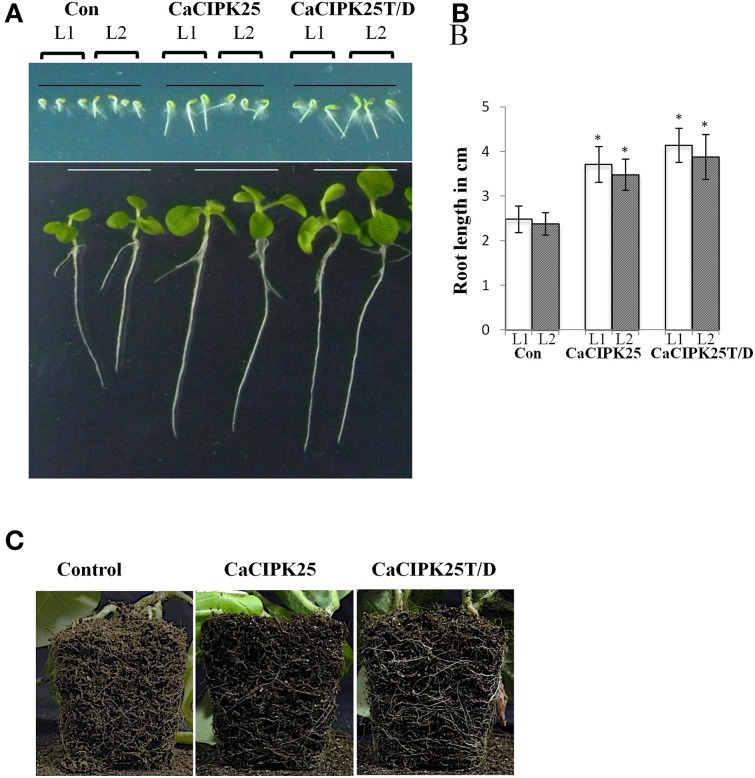
**Morphology of CaCIPK25 overexpressing transgenic tobacco lines**. **(A)** Morphological comparison of vertically grown tobacco seedlings transformed with empty vector (Con), *CaCIPK25* and *CaCIPK25T*^171^*D* genes. Transgenic seeds from two lines (L1 and L2) are represented for all the constructs, upper and lower panels are showing 2− and 15−days old seedlings after germination, respectively. **(B)** Comparison of root lengths of 15-days old seedlings. Averages of three measurements of 15 seedlings each are shown. ^*^ indicates statistically significant difference (*p* < 0.05) from the control sample. **(C)** Root system of soil-grown 50 days-old transgenic tobacco plants. Roots exposed to the soil surface are shown.

To investigate *in planta* function, CaCIPK25 and CaCIPK25T171D (henceforth referred to as CaCIPK25T/D) were expressed in tobacco plants under the control of 35S promoter. More than 10 transgenic lines were raised for each construct along with the lines harboring only pBI121 (vector-control) using the *Agrobacterium*-mediated transformation method. T_3_ homozygous plants were selected for experiments on the basis of expression analysis by RT-PCR (Supplementary Figure 1). Seeds of two individual lines for each construct along with the vector-control seeds were sown on ½MS-agar plates for germination. No difference was observed among the lines with respect to the percentage of and time taken for germination, however, all the lines expressing CaCIPK25 or CaCIPK25T/D displayed longer root length within 2 days of germination. After 15 days, the primary roots of the CaCIPK25− and CaCIPK25T/D-overexpressing lines were 52 and 60% longer than the roots of the control line (Figures 6A,B). The difference in root morphology was more evident in the matured plants. The CaCIPK25− and CaCIPK25T/D− overexpressing plants showed an enlarged root system as compared to the control plants when grown in soil for 50 days (Figure 6C). Although, the leaves of the seedlings expressing both the CaCIPK25 constructs were larger than those of the control seedlings at the early stages after germination probably due to longer root, there was no significant difference in leaf sizes observed between the control and CaCIPK25-expressing tobacco plants in the later stages of growth (Supplementary Figure 2).

### Improved salinity and water deficit tolerance in CaCIPK25-overexpressing plants

**Figure 7:**
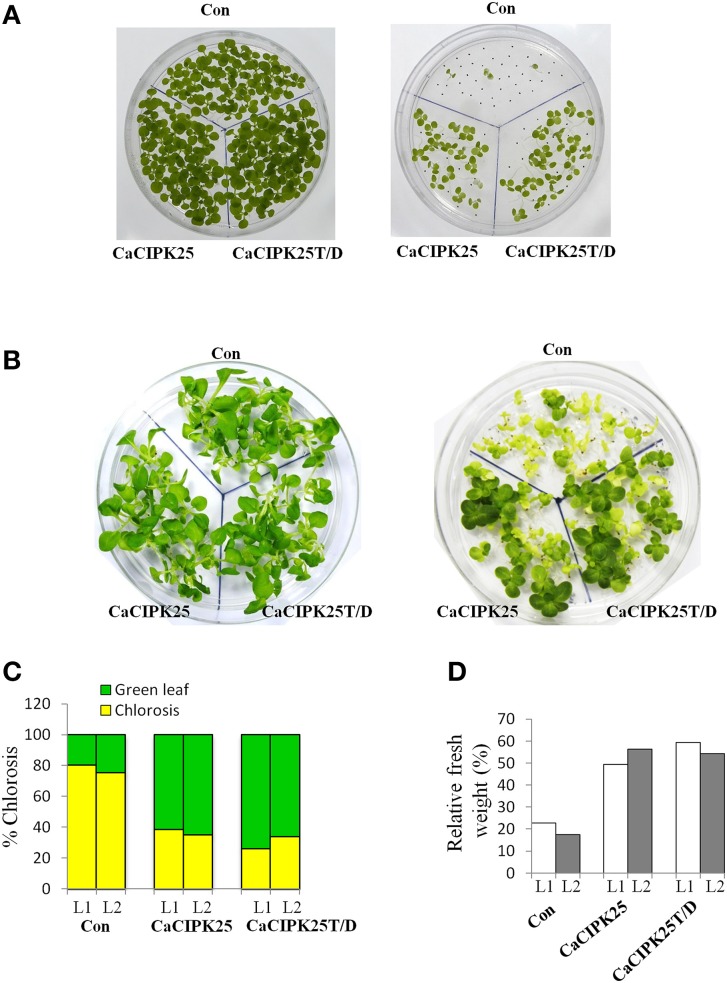
**Effect of salt stress on transgenic tobacco seedlings**. **(A)** Control (vector only), *CaCIPK25*- and *CaCIPK25T/D*- expressing tobacco seeds were germinated on media without (left) and with (right) 200 mM sodium chloride for 15 days. **(B)** 8-day-old seedlings of control, CaCIPK25− and CaCIPK25T/D-expressing lines were transferred to media without (left) and with (right) 250 mM sodium chloride for 10 days and then transferred to normal medium for 8 days. **(C)** Leaves of the seedlings of two representative transgenic lines (L1 and L2) exposed to salt were scored for chlorosis and bleaching and presented as relative to total number of leaves. **(D)** Fresh weights of the seedlings from the same experiment were presented relative to the fresh weights of corresponding seedlings grown in normal medium for the same period.

**Figure 8:**
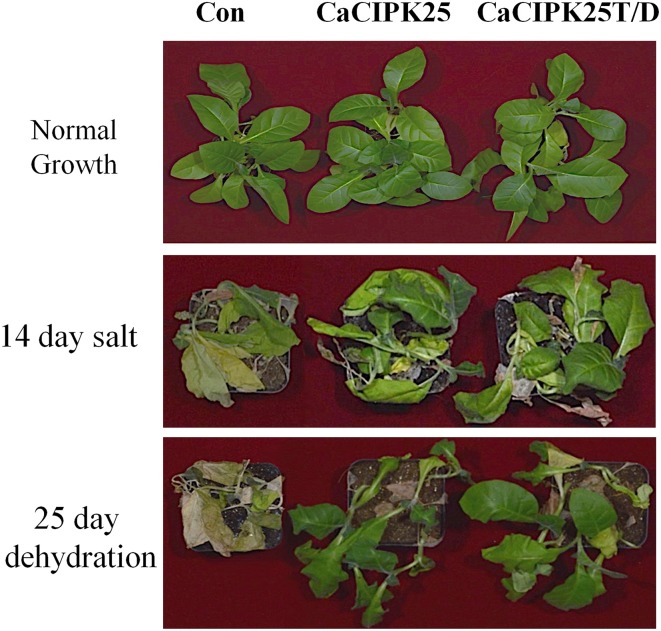
**Effect of salt on transgenic tobacco plants**. Fifty-day-old soil-grown vector-control, *CaCIPK25*- and *CaCIPK25T/D*- overexpressing tobacco plants **(top)** were irrigated with 300 mM sodium chloride for 2 weeks **(middle)** or, not irrigated with water for 25 days **(bottom)**.

To investigate the effect of the CaCIPK25 expression on germination efficiency of the transgenic seeds under high salinity condition, seeds were sown on normal growth medium supplemented with 200 mM sodium chloride and allowed to germinate for 15 days. The tobacco seeds transformed with the empty vector and both the constructs of CaCIPK25 germinated simultaneously and showed similar growth in normal growth medium. Both the CaCIPK25-transformed seeds took 6 days more to germinate on the salt-supplemented medium as compared to their germination on the normal growth medium. Only 7% of vector-control seeds germinated in comparison to 60 and 64% seed germination of CaCIPK25− and CaCIPK25T/D-transformed lines, respectively, after 15-day exposure on high-salt medium (Figure 7A). To assess salt-tolerance of the seedlings, 8-day-old seedlings were exposed to medium supplemented with 250 mM sodium chloride. After 10 days of exposure, the seedlings were transferred to the normal growth medium for recovery for 8 days (Figure 7B). Leaves were scored for chlorosis and the relative fresh weights for each line were assessed by comparing the fresh weights of the corresponding lines continuously grown on the normal growth medium. Approximately 80% leaves of the control plants have undergone chlorosis in contrast to about 36 and 26% chlorosis in the lines expressing CaCIPK25 and CaCIPK25T/D, respectively (Figure 7C). The relative fresh weight of the control seedlings grown in high salinity was about 20% of those grown in normal medium, while the relative fresh weights of the CaCIPKL25− and CaCIPK25T/D− expressing plants were 53 and 57%, respectively (Figure 7D). Enhanced tolerance to salt and drought was also observed in plants grown in pots. Fifty days-old plants of one line for each of the constructs, three in a pot in duplicate, were irrigated with 300 mM sodium chloride twice a week for 2 weeks. All the vector-control plants were etiolated, displayed severe chlorosis and died. The plants expressing both the forms of CaCIPK25 showed a moderate level of chlorosis and etiolation. Similarly, 50-day-old plants in pots were not irrigated for 25 days. All the control plants died within this period while all the plants expressing the either form of CaCIPK25 showed a moderate level of etiolation (Figure 8) and nine out of 12 plants recovered upon further irrigation for 2 weeks.

**Figure 9:**
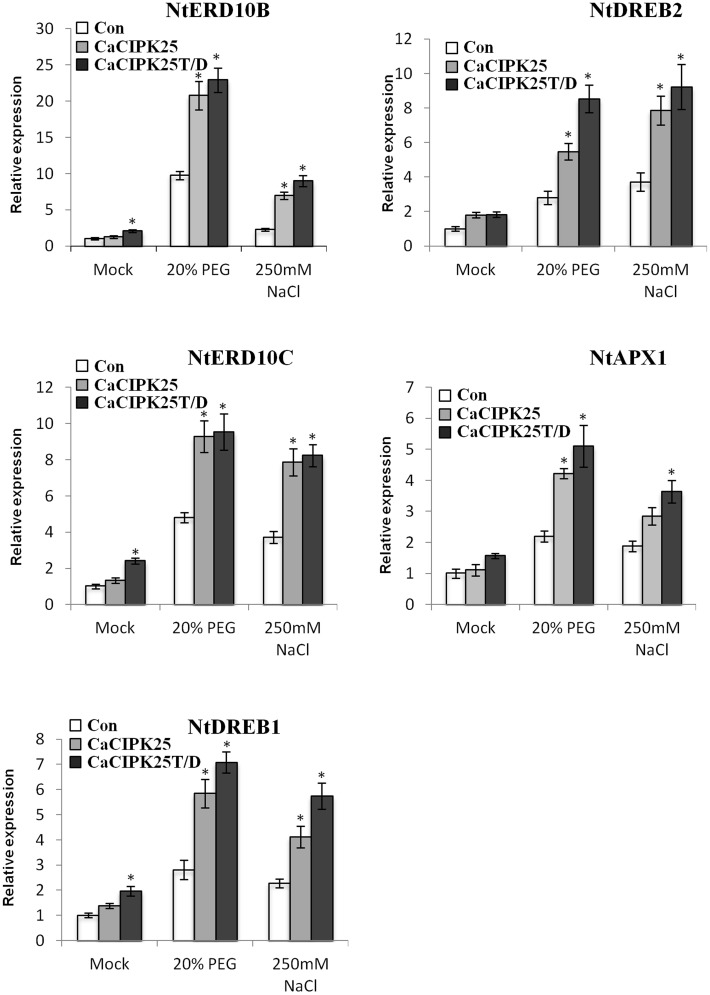
**Expression analysis of abiotic stress marker genes in CaCIPK25 overexpressing plants**. Expression of known abiotic stress marker genes determined by qRT-PCR in 20-day-old control, *CaCIPK25*− and *CaCIPK25T/D*− overexpressing tobacco plants in response to treatments mentioned. Y-axis described the fold change of expression. Tobacco actin gene was used as internal control. ^*^ indicates statistically significant difference (*p* < 0.05) from the control sample.

Expression levels of the known genes related to abiotic stress signaling and tolerance were assessed in the transgenic plants under control and stress treatments. Five genes, namely NtERD10B (GenBank:AB049336), NtERD10C (AB049337), NtDREB1 (EU727155), NtDREB2 (EU727156), NtAPX1 (U15933.1), and were selected on the basis of their reported enhanced expression in transgenic plants showing tolerance to such stresses (Shukla et al., 2006; Tripathi et al., 2009; Bao et al., 2015; Zhou et al., 2015). NtERD10B and NtERD10C encode dehydrins, NtDREB1 and NtDREB2 encode dehydration responsive element (DRE)/C-repeat element (CRE) binding proteins and are transcription factors, and NtAPX1 encode ascorbate peroxidase. Only NtERD10B and NtERD10C showed more than 2-fold increase in expression level with respect to the vector-control plants only in the CaCIPK25T/D-overexpressing line in the control condition. Three other genes showed less than 2-fold increase in this line. Expression of all the five genes was not significantly increased in the CaCIPK25-overexpressing plants. Upon treatment with 20% PEG or 250 mM sodium chloride, expression of these genes increased several folds in the vector-transformed plants. In the CaCIPK25- or CaCIPK25T/D-expressing tobacco lines, increase in expression level of all the five genes was more than 2-fold as compared to the vector-transformed plants when exposed to PEG and sodium chloride. Further, expression of all the five genes were always higher in the CaCIPK25T/D lines than that in the CaCIPK25 lines, suggesting that the higher kinase activity of the protein resulted in higher expression of stress-related genes and, thereby, further enhanced tolerance level (Figure 9).

## Discussions

In this report, we proposed CaCIPK25, a chickpea ortholog of Arabidopsis CIPK25, as a positive regulator of root development and tolerance to water deficit and high salinity. This is the first report on the function of CIPK25 from any plant. This gene was cloned from one of the two ESTs identified in a screening of dehydration-induced ESTs in chickpea. Under normal growth condition, CaCIPK25 expression is restricted to root and flower. Expression of a reporter gene driven by the 5′-UAS of CaCIPK25 in Arabidopsis also supported this expression pattern, suggesting that similar factors controlled this promoter activity in these two plants. The expression pattern surprisingly corresponded to that of the rolD gene of *Agrobacterium rizogenes*. CaCIPK25 promoter, like that of rolD, possesses multiple copies of similar root− and flower-specific *cis*-acting elements. Infection with *A. rhizogenes* with mutated *rolD* gene resulted in attenuated root growth and formation of callus (White et al., 1985). A fine balance of auxin and cytokinin concentrations in root regulates natural root growth. While auxin promotes cell division at the root apical meristem, cytokinin promotes cell differentiation at the elongation zone of the root (Chapman and Estelle, 2009). Therefore, it appears that rolD functions to maintain the balance between auxin and cytokinin and, thereby, promotes root growth. Antagonistic expression pattern of CaCIPK25 in response to auxin and cytokinin, absence of its expression at the root apex and lateral root initials having high auxin concentration and enhanced root growth in the overexpressing lines indicates its involvement in maintaining balance between auxin and cytokinin. This was further supported by the presence of auxin-responsive and cytokinin-responsive *cis*-acting elements in the 5′-UAS of the gene. The other organ that showed high CaCIPK25 expression was the flower. CaCIPK25 expression in flower was growth stage dependent, similar to that of rolD. Transgenic tobacco plants expressing rolD were early flowering and displayed earlier and enhanced organogenesis of flowers (Mauro et al., 1996). We did not observe any apparent differential morphology or period taken for flowering. Most probably, enhanced expression of CIPK25 alone was not enough to bring about altered morphology in the reproductive organs. On the other hand, CIPK25 activity in reproductive tissues was already saturated and, therefore, overexpression could not alter the morphology or period taken for flowering.

CaCIPK25 overexpressing plants showed enhanced root growth in normal growth condition. It suggested that there was a potential for the root to grow longer. CIPK25 activity for root growth was limiting and overexpression of the protein must activated a signaling pathway that regulates root growth. Overexpression of high active CaCIPK25 mutant (CaCIPK25T/D) caused a further increase in the root length suggesting the kinase activity was required for this function. The larger leaf size of the CaCIPK25-overexpressing seedlings with respect to the control seedlings at the early stage of growth was most probably due to faster root growth and, thereby, absorption of more nutrient from the medium. The shoot size of the control plants was recovered at the later stage. This result and a very low expression of *CaCIPK25* in stem and leaf suggested that associated factors required for CaCIPK25 to promote shoot growth were absent. Fold increases in the marker gene expression in CaCIPK25T/D-overexpressing plants were higher than those in the CaCIPK25-overexpressing plants. Higher fold increase in the marker gene expression and in the limit of tolerance in the *CaCIPK25T/D*-overexpressing plants suggested that the full potential of CaCIPK25 activity was not achieved even after stress treatment. We assessed the expression level of a few marker genes related to abiotic stress tolerance. Expression of only NtERD10B and NtERD10C was increased by 2-fold in the CaCIPK25T/D-overexpressing lines as compared to the vector-control plant in normal growth condition. Expression of all the marker genes increased many folds only after stress treatment. This result suggested that just overexpression of CaCIPK25 alone or longer root length was not enough for introducing stress tolerance. It appeared that stress tolerance mechanism of CaCIPK25-overexpressing plants was activated only after stress treatment. Similar observation was previously reported in case of *Arabidopsis* DREB2A protein, where stress treatment was required to nullify the negative regulatory effect of a negative element in the DREB2A protein (Sakuma et al., 2006). There are several reports demonstrating the importance of DREB protein family in drought and salinity tolerance (Agarwal et al., 2006; Morran et al., 2011; Jiang et al., 2014). CaCIPK25 overexpression caused enhanced expression of tobacco DREB1 and DREB2. A previous report described that the overexpression of a calcium binding peptide CBP in *Arabidopsis* resulted in the increase in total Ca^+2^ store in the cell and provided salinity tolerance to the plant. CBP-overexpressing plants showed higher expression of DREB1A and CIPK6. However, when crossed with cipk6 mutant, the CBP-overexpressing plants did not show any enhancement in the salinity tolerance and expression of DREB1A, suggesting CIPK6 was involved in Ca^+2^-mediated expression of DREB1A (Tsou et al., 2012). This observation, and the enhanced expression of tobacco DREB1 and DREB2 in the CaCIPK25-overexpressing plants after stress treatment indicated that there might be some connections between the CIPKs and DREBs in the stress-regulated Ca^+2^-mediated signaling. Cytosolic Ascorbate peroxidase1 (APX1) was previously reported to play a key role in the stress acclimation of *Arabidopsis* (Koussevitzky et al., 2008).

In this report, we have presented an expression analysis of chickpea *CaCIPK25* gene and characterized the transgenic tobacco plants overexpressing this gene. *CaCIPK25* expressed preferentially in the root, except in root apex, and flower. Its overexpression in tobacco plants resulted in enlarged root system with a normal shoot morphology. Although, highly expressed in flower, the overexpressing plant did not show any apparent morphological disorder in the reproductive organ and the flowers were fertile. Overexpression of *CaCIPK25* in tobacco enhanced the tolerance of the transgenic plant to water deficit and high salinity. Replacement of a conserved threonine residue with aspartic acid in the activation domain of the protein increased its kinase activity. Overexpression of this active kinase caused further enhancement of root growth and tolerance to stress treatments as compared to that of the wild-type protein suggesting that the kinase activity was important for these functions. Altogether, we have reported for the first time a functional characterization of CIPK25 from a plant.

## Author contributions

MM, SG, VD, and AR conducted the experiments, interpreted the results, and prepared the first draft. DC conceptualized the study, designed the experiments, interpreted the results and made the final draft. All the authors approved the final version.

### Conflict of interest statement

The authors declare that the research was conducted in the absence of any commercial or financial relationships that could be construed as a potential conflict of interest.
