# Supplementary material for: Expression of chickpea CIPK25 enhances root growth and tolerance to dehydration and salt stress in transgenic tobacco
Source: Front Plant Sci. 2015 Sep 8;6:683. doi: 10.3389/fpls.2015.00683 (PMC4561800; doi:10.3389/fpls.2015.00683)
Supplement: Supplementary file 1 [file Image1.PDF]

# Supplementary Figures

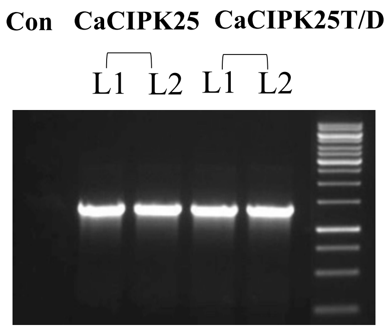


Supplementary Figure 1. Expression of *CaCIPK25* transgene in the T3 transgenic lines assessed by RT-PCR. Extreme right panel shows size ladder.

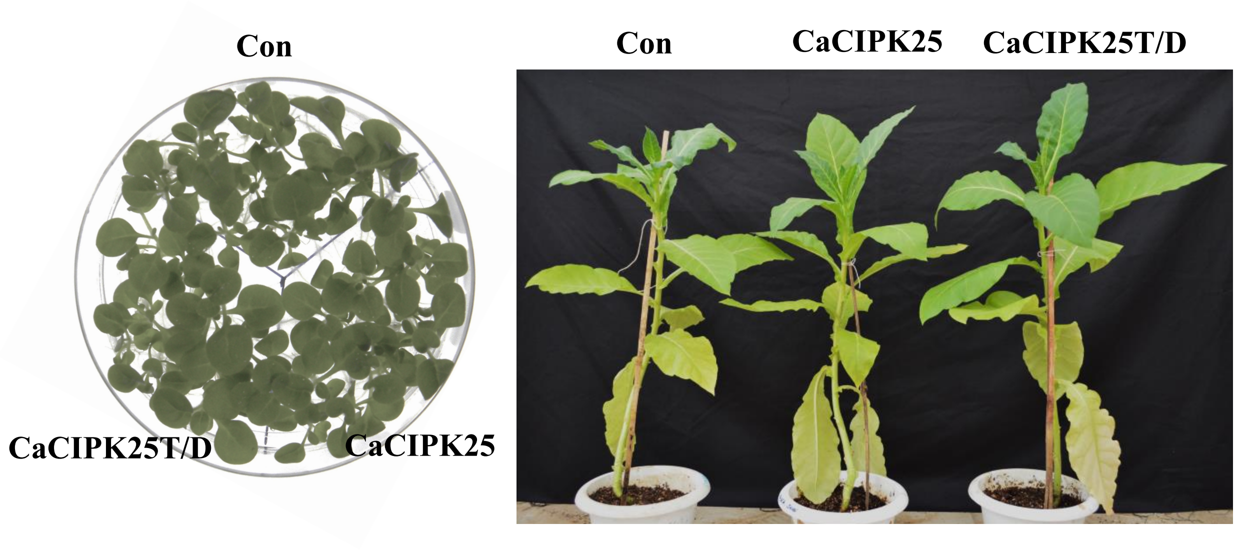


Supplementary Figure 2. Phenotype of 30 day-old plate-grown and 75 day-old soil-grown transgenic tobacco lines harboring empty vector (Con), *CaCIPK25* and *CaCIPK25T/D*.
