# Supplementary material for: Expression of chickpea CIPK25 enhances root growth and tolerance to dehydration and salt stress in transgenic tobacco
Source: Front Plant Sci. 2015 Sep 8;6:683. doi: 10.3389/fpls.2015.00683 (PMC4561800; doi:10.3389/fpls.2015.00683)
Supplement: Supplementary file 2 [file Image2.PDF]

Supplementary Tables

Supplementary Table1: Primers sequences used in *CaCIPK25* cloning

Name of primer	Sequence of primers
5’ RACE F1	5’-ATATATCTCCGCCGTCACAGCCAACTTC-3’
5’ RACE F2	5’-AGCCAACTTCCCTTTTCTCCCTTCCATC-3’
5’ RACE F3	5’-ACCCGATAGATCAAACCCCGAAGACATC-3’
3’ RACE R1	5’-GGGATGATGGAAGGGAGAAAAGGGAAG-3’
3’ RACE R2	5’-AAGGGAGAAAAGGGAAGTTGGCTGTGAC-3’
3’ RACE R3	5’-GGCCTGCTCTCAAAGACATTGTTTGGTC-3’
CaCIPK25F XbaI	5’- GCTCTAGAATGGAGGAATTAAAAGCAAAC-3’
CaCIPK25R SacI	5’- GCGAGCTCTCATTCCCCCTGCCATGAC-3’
CaCIPK25pr F3	5’-CCAAGCTTCTCTTAGAATGGTTAATAATGTATGACA-3’
CaCIPK25pr R3	5’- CGGGATCCTGGAAAATAAAAATAAAAACAGTAGCAC-3’
CaCPK25prdel1 F	5’- CCAAGCTTATCAATTGTAGGCGGAGGCGA-3’
CaCPK25prdel2 F	5’- CCAAGCTTTTCGAGGGAGGAGAGGACAAAC-3’
CaCIPK25 EcoRI F	5’- CGGAATTCATGGAGGAATTAAAAGCAAAC-3’
CaCIPK25 NotI R	5’- ATGCGGCCGCTCATTCCCCCTGCCATGAC-3’
CaCIPK25 T/D171F	5’- GACGGGCTTTTACATGATCAATGTGGGACC-3’
CaCIPK25 T/D171R	5’- GGTCCCACATTGATCATGTAAAAGCCCGTC-3’

Supplementary Table2: Primers sequences used in qRT-PCR

Name of primer	GenBank Accession no.	Sequence of primers
EF1a RealT_F	AJ004960.1	5’-TCCACCACTTGGTCGTTTTG-3’
EF1a RealT_R		5’-CTTAATGACACCGACAGCAACAG-3’
CaCIPK25 RT F	XM_004498761.1	5’- GGCCTGCTCTCAAAGACATT-3’
CaCIPK25 RT R		5’- CGGTACACGTGGCATCATT-3’
Ntactin_RT F	BAD27408	5’-ATCCATGAGACTACCTACAACT-3’
Ntactin_RT R		5’-CCACCACTGAGCACAATG-3’
NtDREB1_RT F	EU727155	5’-GGTTACATTAGGCGAAGAG-3’
NtDREB1_RT R		5’-TTCTCAGACGAACTCCTC-3’
NtDREB2_RT F	EU727156	5’-GAAACGCCAGAAAGTAGT-3’
NtDREB2_RT R		5’-ATTAGTCCTTCCGCCATA-3’
NtERD10B_RT F	AB049336	5’-CGGACGAATACGGCAATC-3’
NtERD10B_RT R		5’-CAGCGTGAGTTCCATAGG-3’
NtERD10C_RT F	AB049337	5’- ACGAGCTTAGCAACAAGTT -3’
NtERD10C_RT R		5’- GGAAATCAAACAAACCACGA -3’
NtAPX _RT F	U15933.1	5’- TGAGGCTCACTTGAAGCTCT-3’
NtAPX _RT R		5’- CACTCCCAACTCTTCCTCCT-3’
