# Supplementary material for: Expression of chickpea CIPK25 enhances root growth and tolerance to dehydration and salt stress in transgenic tobacco
Source: Front Plant Sci. 2015 Sep 8;6:683. doi: 10.3389/fpls.2015.00683 (PMC4561800; doi:10.3389/fpls.2015.00683)
Supplement: Supplementary file 3 [file Image3.PDF]

Supplementary Text

> CaCIPK25 cDNA/gene sequence (1727bp). Translation start and stop codons are shown in bold font

CCCACTTCCATCACACAACCAACATTCCTTCACTCCTCTTCTTCCTTTCCTTAGCCTAACTT
CTCAACAACCTTCCAAAATTCCCACACAAAAAAATGTCCTTACAAAATAACCCGTTAATC
ATGCTCTAGTCACTACCATACTTTGTGCTACTGTTTTTATTTTTATTTTCCA**ATG**GAGGAA
TTAAAAGCAAACATATTATTTGGAAAATATGAGATGGGAAGGGTACTAGGCAAAGGAAC
ATTCGCAAAAGTATACTATGGAAAAGAAATTGAAACTGGTGAAGGAGTAGCAATAAAAG
TAATCGACAAAAACAAAGTGaAAAAAGAAGGAATGATGGAGCAAATCAAACGCGAGAT
TTCCGTGATGAGGTTAGTAAAACATCCAAACATCGTGAACCTTAAAGAAGTCATGGCAA
CaAAAACAAAAATCTTGTTCGTGATGGAATACATACGTGGCGGCGAATTATTCGCCAAAG
TGGAAAAGGGAAAATTAAAAGAAGAAACCGCTAGAAAGTACTTCCAACAACTCATAAG
CGCGGTTGAATTTTGCCACAGTAGAGGAGTTGCACACCGCGATTTAAAACCTGAGAATTT
GTTACTCGACGAACACGGAGACCTTAAAATCTCCGATTTTGGTCTCTCCGCTTTGCCAGA
ACAGCTTCGGCAGGACGGGCTTTTACATACTCAATGTGGGACCCCCGCTTACGTGGCACC
CGAGGTTGTTAGAAAAAGAGGTTATAACGGTTTTAAAGCTGATACTTGGTCTTGTGGTGT
TATTCTTTATGCTTTACTTGCTGGATTTCTTCCTTTTCAACATGAAAATCTTATTACTATGT
ATAATAAGGTTTTCAAAGAAGAGTATCGATTCCCACCTTGGTTTTCGCCTGAATCAAAGA
GATTAATCTCGAAGATTCTAGTTGCGGATCCTGAAAGAAGAATCACTATTTCTTCAATTA
TAAATGATACATGGTTCAAAAAAGGTTTATCAACTATTAATTCAAATGATGATTTGGAAT
CAGAGATGGTAATGATTAATTCTTCGCCTAAATTCTTCAATGCGTTTGAATTTATTTCTTC
GATGTCTTCGGGGTTTGATCTATCGGGTTTATTCGAGGAAAAGaAAAAGAGAGGTTCTGT
TTTTACTTCCAAGTGTTCTGTTTCGGAGATTGTGTCTAAGATTGAAAGTGCTGCGAAAAG
TTTAAGGTTTAAGGTTGGGAAGGTTAAGGAATTTAAATTGAAGTTGCAAGGGATGATGG
AAGGGAGAAAAGGGAAGTTGGCTGTGACGGCGGAGATATATGAGGTGGCGCCGGAGCT
TGCGGTGGTTGAATTTTCTAAATGTTCCGGTGATACCTTTGAATATGTCAAGTTTTTTGAA
GATGATGTTAGGCCTGCTCTCAAAGACATTGTTTGGTCATGGCAGGGGGAA**TGA**TGCCA
CGTGTACCGGTAACATGGCTAATTAAGATTCTCTTAATTTTGGGTGATAATATTAGTATTT
TATGATTTCTCTGAAATTTGTGTATATAATTTTAATATATGCTTAGAGTTGGGTGCTTCTA
GTTGAATAAGTTCTAACTAACTTTTCCTTCTCTTTTTTCACTTTTTGTGCATTTACTAGTGG
CTTGAATGTTATGCAGATAGTTAGGATTTTGGTGAGACTGTACATACTCCACCCTAGAAG
CCCTCGATTGTATAATAATAAAGTTAATTTTGAGGAAGAAATAACAA

>CaCIPK25 protein (418aa)

MEELKANILFGKYEMGRVLGKGTFAKVYYGKEIETGEGVAIKVIDKNKVKKEGMMEQIKRE
ISVMRLVKHPNIVNLKEVMATKTKILFVMEYIRGGELFAKVEKGKLKEETARKYFQQLISAV
EFCHSRGVAHRDLKPENLLLDEHGDLKISDFGLSALPEQLRQDGLLHTQCGTPAYVAPEVVR
KRGYNGFKADTWSCGVILYALLAGFLPFQHENLITMYNKVFKEEYRFPPWFSPESKRLISKIL
VADPERRITISSIINDTWFKKGLSTINSNDDLESEMVMINSSPKFFNAFEFISSMSSGFDLSGLFE
EKKKRGSVFTSKCSVSEIVSKIESAAKSLRFKVGKVKEFKLKLQGMMEGRKGKLAVTAEIYE
VAPELAVVEFSKCSGDTFEYVKFFEDDVRPALKDIVWSWQGE

5'UAS (promoter) of CaCIPK25

>CaCIPK25 promoter

ATATT = ROOTMOTIFTAPOX1

AGAAA= POLLEN1LELAT52

The sequences bordering the promoter deletion constructs are marked by red fonts.

-2196
CTCTTAGAATGGTTAATAATGTATGACATTAGTTTATTGTAAGAGTACATTTTATTGTTTA
TAAAAAATTAACATCATACACTATTGATTTGAATATTAATTATTTTAAACATTATCATATA
TAATTTTAAAAAATTAAATTATTTGTTAATATAGGTATTTCTTATATTTGACAAACGTTGA
AGTATATTTAGTGATTTTGGTTCTTTAAAATTTAAACTAAAATACACATATTATTATGTGT
ATACATAATTTCCTTTAGAGATTTTAAGATTTTTGTTTTTCTATTATTTTTGAATTTATAAA
TCTCAATTAGTAAAATAGTTCAATAAAATTGACTTTCTGTTATTAGATATTTTTATTATTT
GACAAATATAATAACATATTTGTAAAATATGTTTTTCCTAAATTTGAAATTGAAGATGAC
AATTATTATGTGTATGTATATATTATACGTACATGTTTCTCTTAAACTCTTTAATCGACTA
TTATGCATAAATTTATGAATATATTTTGATTAATGATCTGAAAATTTTAAAATTTGGATTT
TTTTTAAATTTTGTAAAGTTTATAATATATCTTTAAAGATTTTGTAATTTTTATTTGAGTTA
TTTTGAAAAAAATATGACTGAAAAAATAGTGAATTAAAAAATTTTATATTATGAAAAAT
AAATACATAAGGGGGAGGAATTAAGAAAATAACTTATTTTATGAAGATGTAATAATTTT
AAATTTTATATATAGAATCTTTTTGAATTATTTCAATTTCGTTAAATTCCTTATCTAGATTT
TTTATTTATTTAAATTATATTATCTCAATTGAGTTCTATATAAGTAACAAAAAGCATATGG
TTTTAAGGTTGAATGATTAAAAAAAATATAAATTTTAGAATATAATATTTAAATTTTTAG
AATAAAAAATATTATAAAAAATAAAAATGAAGACATAAGGGGAAGGCATTAAAAATAT
AAAAGTAAAAACAAAAATTTATTTGTAAAAGTGGAAAAATTGAAATTTAAAATTTAAAA
TCTTTTTAGATTATTTCCATTCAATTAAATTCTTTATTTAGATTGCTTTTTTTAAAAAAAAT
TTAAATAATAATATTTTAATTAAATTATATATGAATGCTGAATGATTATGGTAAAATA**AT
CAAT**TGTAGGCGGAGGCGAAATTTTTATTTCATAAAATCGGTTAATTATATAATTTTTTTA
GTGAAATAATGACAAGAAATTTATGTGGAGAATTTTCTAAGATGGCAATTTTTTTATGAA
AAATATCGTTAGGTGGACTGAACTAATAAAATTGAGGATTGTCCCCGCCAAACAAAACA
GTGCAACCAGACTAGAAGGGATGTACTAACTACTAACTATAAATATTATAATTTCAACAT
TTGATGGACCAAACGTAGGAAACCAAATAATTTATATTTCATATTTTATCCCTTTATTATG
TGTCTCTACTTTTTTATTTTCTGTTCAAAGAATATATGTTATAAAAAAAAGTCTAACAACT
TAAAAATACTATCGTTGTTAGCAAGAAAATTAATTGTTATTAAATAGAAACAATGGTTAA
AGGAATGTACCAAAAAAAGAAAACAATATTAAAATCAAAACTGTTAATATTCATAAAGA
AATGGTAAAAAAGAAAAAATAACATAAATTAATATTTCTTCTGTCTCATAATATATTATA
AAAATTAAATATATTTAGTCTAAATTTGGACATATATTTTTATTTAATAGTATCATAGAAA
GATATTATTGAATAAAAAACACATTATATTATTAAATAAAAAACAGAAACAAACAATTT
TCATTTA**TTCGAG**GGAGGAGAGGACAAACAGCCCACAAATTCAATCTAAGATCCGTGGG
CTTGAATTCTTCCAAAGCAAAAAAAATCAAAACAACAAACAAATGTCTCTAATGATAAT
GAGTTAACTAGTTAGTCGACCAAAACTTCCTTCTCACCTCTATTATTAATTGCATTCTTTA
ATTTCATTATTAGAAGGGGTCCCTCCCAACAAGGCTCCCAACTTTAACGTGGCTTTCCATT
TCACCTATATAATTATAATTTAATCACATTTCAATTCCGTGTGCTCTCTAATTCAAAACTA
ACAAGTGTTCCAATGGTTGCCAGCCAAACCGTCACTCCTCTTCCCTTTATCACTACCTTTA
ATTAAACCCAACCCAAACCAAA-1

In silico analysis of CaCIPK25 promoter /5'UAS

Factor or Site Name		Loc.	(Str.)	Signal Sequence	SITE #
-10PEHVPSBD	site	889	(-)	TATTCT	S000392
-10PEHVPSBD	site	911	(-)	TATTCT	S000392
-10PEHVPSBD	site	1481	(-)	TATTCT	S000392
-300CORE	site	561	(+)	TGTAAAG	S000001
-300ELEMENT	site	388	(+)	TGHAAARK	S000122
-300ELEMENT	site	561	(+)	TGHAAARK	S000122
-300ELEMENT	site	663	(+)	TGHAAARK	S000122
-300ELEMENT	site	994	(+)	TGHAAARK	S000122
-300ELEMENT	site	1269	(+)	TGHAAARK	S000122
AACACOREOSGLUB1	site	1801	(+)	AACAAAC	S000353
AACACOREOSGLUB1	site	1905	(+)	AACAAAC	S000353
ABRELATERD1	site	2038	(+)	ACGTG	S000414
ABRERATCAL	site	2037	(+)	MACGYGB	S000507
ACGTABOX	site	453	(+)	TACGTA	S000130
ACGTABOX	site	453	(-)	TACGTA	S000130
ACGTABREMOTIFA2OSEM	site	2038	(+)	ACGTGKC	S000394
ACGTATERD1	site	177	(+)	ACGT	S000415
ACGTATERD1	site	454	(+)	ACGT	S000415
ACGTATERD1	site	1404	(+)	ACGT	S000415
ACGTATERD1	site	2038	(+)	ACGT	S000415
ACGTATERD1	site	177	(-)	ACGT	S000415
ACGTATERD1	site	454	(-)	ACGT	S000415
ACGTATERD1	site	1404	(-)	ACGT	S000415
ACGTATERD1	site	2038	(-)	ACGT	S000415
ACGTTBOX	site	176	(+)	AACGTT	S000132
ACGTTBOX	site	176	(-)	AACGTT	S000132
AMYBOX1	site	836	(+)	TAACARA	S000020
AMYBOX1	site	144	(-)	TAACARA	S000020
AMYBOX1	site	342	(-)	TAACARA	S000020
ANAERO1CONSENSUS	site	979	(+)	AAACAAA	S000477
ANAERO1CONSENSUS	site	1322	(+)	AAACAAA	S000477
ANAERO1CONSENSUS	site	1800	(+)	AAACAAA	S000477
ANAERO1CONSENSUS	site	1908	(+)	AAACAAA	S000477
ANAERO1CONSENSUS	site	276	(-)	AAACAAA	S000477
ARFAT	site	1453	(+)	TGTCTC	S000270
ARFAT	site	1674	(+)	TGTCTC	S000270
ARFAT	site	1915	(+)	TGTCTC	S000270
ARR1AT	site	263	(+)	NGATT	S000454
ARR1AT	site	271	(+)	NGATT	S000454
ARR1AT	site	586	(+)	NGATT	S000454
ARR1AT	site	786	(+)	NGATT	S000454
ARR1AT	site	1037	(+)	NGATT	S000454
ARR1AT	site	1069	(+)	NGATT	S000454
ARR1AT	site	544	(+)	NGATT	S000454
ARR1AT	site	1308	(+)	NGATT	S000454
ARR1AT	site	86	(+)	NGATT	S000454
ARR1AT	site	195	(+)	NGATT	S000454
ARR1AT	site	515	(+)	NGATT	S000454
ARR1AT	site	866	(+)	NGATT	S000454
ARR1AT	site	1134	(+)	NGATT	S000454
ARR1AT	site	305	(-)	NGATT	S000454
ARR1AT	site	480	(-)	NGATT	S000454
ARR1AT	site	745	(-)	NGATT	S000454
ARR1AT	site	1028	(-)	NGATT	S000454
ARR1AT	site	1149	(-)	NGATT	S000454
ARR1AT	site	1187	(-)	NGATT	S000454
ARR1AT	site	1606	(-)	NGATT	S000454
ARR1AT	site	1855	(-)	NGATT	S000454
ARR1AT	site	1896	(-)	NGATT	S000454
ARR1AT	site	2074	(-)	NGATT	S000454
ASF1MOTIFCAMV	site	2143	(-)	TGACG	S000024
ATHB6COREAT	site	427	(+)	CAATTATTA	S000399
BIHD1OS	site	24	(-)	TGTCA	S000498
BIHD1OS	site	171	(-)	TGTCA	S000498
BIHD1OS	site	367	(-)	TGTCA	S000498
BIHD1OS	site	424	(-)	TGTCA	S000498
BIHD1OS	site	1222	(-)	TGTCA	S000498

BOXIINTPATPB	site	741	(+)	ATAGAA	S000296
BOXIINTPATPB	site	1557	(+)	ATAGAA	S000296
BOXIINTPATPB	site	1747	(+)	ATAGAA	S000296
BOXIINTPATPB	site	283	(-)	ATAGAA	S000296
BOXIINTPATPB	site	825	(-)	ATAGAA	S000296
BOXIIPCCHS	site	2038	(+)	ACGTGGC	S000229
CAATBOX1	site	310	(+)	CAAT	S000028
CAATBOX1	site	327	(+)	CAAT	S000028
CAATBOX1	site	427	(+)	CAAT	S000028
CAATBOX1	site	763	(+)	CAAT	S000028
CAATBOX1	site	817	(+)	CAAT	S000028
CAATBOX1	site	1051	(+)	CAAT	S000028
CAATBOX1	site	1152	(+)	CAAT	S000028
CAATBOX1	site	1258	(+)	CAAT	S000028
CAATBOX1	site	1564	(+)	CAAT	S000028
CAATBOX1	site	1597	(+)	CAAT	S000028
CAATBOX1	site	1807	(+)	CAAT	S000028
CAATBOX1	site	1854	(+)	CAAT	S000028
CAATBOX1	site	2084	(+)	CAAT	S000028
CAATBOX1	site	2124	(+)	CAAT	S000028
CAATBOX1	site	36	(-)	CAAT	S000028
CAATBOX1	site	54	(-)	CAAT	S000028
CAATBOX1	site	84	(-)	CAAT	S000028
CAATBOX1	site	334	(-)	CAAT	S000028
CAATBOX1	site	416	(-)	CAAT	S000028
CAATBOX1	site	819	(-)	CAAT	S000028
CAATBOX1	site	1009	(-)	CAAT	S000028
CAATBOX1	site	1071	(-)	CAAT	S000028
CAATBOX1	site	1154	(-)	CAAT	S000028
CAATBOX1	site	1303	(-)	CAAT	S000028
CAATBOX1	site	1310	(-)	CAAT	S000028
CAATBOX1	site	1546	(-)	CAAT	S000028
CAATBOX1	site	1760	(-)	CAAT	S000028
CAATBOX1	site	1979	(-)	CAAT	S000028
CACTFTPPCA1	site	80	(+)	YACT	S000449
CACTFTPPCA1	site	2146	(+)	YACT	S000449
CACTFTPPCA1	site	2164	(+)	YACT	S000449
CACTFTPPCA1	site	2199	(+)	YACT	S000449
CACTFTPPCA1	site	1355	(+)	YACT	S000449
CACTFTPPCA1	site	1362	(+)	YACT	S000449
CACTFTPPCA1	site	1459	(+)	YACT	S000449
CACTFTPPCA1	site	1520	(+)	YACT	S000449
CACTFTPPCA1	site	44	(-)	YACT	S000449
CACTFTPPCA1	site	184	(-)	YACT	S000449
CACTFTPPCA1	site	193	(-)	YACT	S000449
CACTFTPPCA1	site	315	(-)	YACT	S000449
CACTFTPPCA1	site	639	(-)	YACT	S000449
CACTFTPPCA1	site	834	(-)	YACT	S000449
CACTFTPPCA1	site	974	(-)	YACT	S000449
CACTFTPPCA1	site	1000	(-)	YACT	S000449
CACTFTPPCA1	site	1212	(-)	YACT	S000449
CACTFTPPCA1	site	1331	(-)	YACT	S000449
CACTFTPPCA1	site	1741	(-)	YACT	S000449
CACTFTPPCA1	site	2117	(-)	YACT	S000449
CARGCW8GAT	site	48	(+)	CWWWWWWWWG	S000431
CARGCW8GAT	site	163	(+)	CWWWWWWWWG	S000431
CARGCW8GAT	site	1061	(+)	CWWWWWWWWG	S000431
CARGCW8GAT	site	1443	(+)	CWWWWWWWWG	S000431
CARGCW8GAT	site	48	(-)	CWWWWWWWWG	S000431
CARGCW8GAT	site	163	(-)	CWWWWWWWWG	S000431
CARGCW8GAT	site	1061	(-)	CWWWWWWWWG	S000431
CARGCW8GAT	site	1443	(-)	CWWWWWWWWG	S000431
CATATGGMSAUR	site	846	(+)	CATATG	S000370
CATATGGMSAUR	site	846	(-)	CATATG	S000370
CBFHV	site	481	(+)	RYCGAC	S000497
CBFHV	site	1945	(+)	RYCGAC	S000497
CBFHV	site	1945	(-)	RYCGAC	S000497
CCA1ATLHCB1	site	271	(-)	AAMAATCT	S000149
CCA1ATLHCB1	site	786	(-)	AAMAATCT	S000149
CCAATBOX1	site	2123	(+)	CCAAT	S000030
CEREGLUBOX3PSLEGA	site	994	(+)	TGTAAAAGT	S000034
CIACADIANLELHC	site	1754	(-)	CAANNNNATC	S000252
CPBCSPOR	site	347	(+)	TATTAG	S000491
CPBCSPOR	site	1999	(+)	TATTAG	S000491
CPBCSPOR	site	1295	(-)	TATTAG	S000491
CRTDREHVCBF2	site	1945	(+)	GTCGAC	S000411
CRTDREHVCBF2	site	1945	(-)	GTCGAC	S000411

CURECORECR	site	45	(+)	GTAC	S000493
CURECORECR	site	456	(+)	GTAC	S000493
CURECORECR	site	1354	(+)	GTAC	S000493
CURECORECR	site	1580	(+)	GTAC	S000493
CURECORECR	site	45	(-)	GTAC	S000493
CURECORECR	site	456	(-)	GTAC	S000493
CURECORECR	site	1354	(-)	GTAC	S000493
CURECORECR	site	1580	(-)	GTAC	S000493
DOFCOREZM	site	564	(+)	AAAG	S000265
DOFCOREZM	site	584	(+)	AAAG	S000265
DOFCOREZM	site	842	(+)	AAAG	S000265
DOFCOREZM	site	972	(+)	AAAG	S000265
DOFCOREZM	site	998	(+)	AAAG	S000265
DOFCOREZM	site	1479	(+)	AAAG	S000265
DOFCOREZM	site	1500	(+)	AAAG	S000265
DOFCOREZM	site	1572	(+)	AAAG	S000265
DOFCOREZM	site	1589	(+)	AAAG	S000265
DOFCOREZM	site	1628	(+)	AAAG	S000265
DOFCOREZM	site	1642	(+)	AAAG	S000265
DOFCOREZM	site	1751	(+)	AAAG	S000265
DOFCOREZM	site	1885	(+)	AAAG	S000265
DOFCOREZM	site	206	(-)	AAAG	S000265
DOFCOREZM	site	257	(-)	AAAG	S000265
DOFCOREZM	site	339	(-)	AAAG	S000265
DOFCOREZM	site	476	(-)	AAAG	S000265
DOFCOREZM	site	580	(-)	AAAG	S000265
DOFCOREZM	site	748	(-)	AAAG	S000265
DOFCOREZM	site	1031	(-)	AAAG	S000265
DOFCOREZM	site	1061	(-)	AAAG	S000265
DOFCOREZM	site	1075	(-)	AAAG	S000265
DOFCOREZM	site	1443	(-)	AAAG	S000265
DOFCOREZM	site	1461	(-)	AAAG	S000265
DOFCOREZM	site	1987	(-)	AAAG	S000265
DOFCOREZM	site	2033	(-)	AAAG	S000265
DOFCOREZM	site	2044	(-)	AAAG	S000265
DOFCOREZM	site	2158	(-)	AAAG	S000265
DOFCOREZM	site	2170	(-)	AAAG	S000265
DPBFCOREDCDC3	site	2115	(-)	ACACNNG	S000292
E2FCONSENSUS	site	1317	(-)	WTTSSCSS	S000476
EBOXBNNAPA	site	817	(+)	CANNTG	S000144
EBOXBNNAPA	site	846	(+)	CANNTG	S000144
EBOXBNNAPA	site	1152	(+)	CANNTG	S000144
EBOXBNNAPA	site	1389	(+)	CANNTG	S000144
EBOXBNNAPA	site	1911	(+)	CANNTG	S000144
EBOXBNNAPA	site	2115	(+)	CANNTG	S000144
EBOXBNNAPA	site	817	(-)	CANNTG	S000144
EBOXBNNAPA	site	846	(-)	CANNTG	S000144
EBOXBNNAPA	site	1152	(-)	CANNTG	S000144
EBOXBNNAPA	site	1389	(-)	CANNTG	S000144
EBOXBNNAPA	site	1911	(-)	CANNTG	S000144
EBOXBNNAPA	site	2115	(-)	CANNTG	S000144
EECCRCAH1	site	337	(+)	GANTTNC	S000494
EMHVCHORD	site	561	(+)	TGTAAAGT	S000452
ERELEE4	site	2102	(+)	AWTTCAAA	S000037
ERELEE4	site	293	(-)	AWTTCAAA	S000037
ERELEE4	site	410	(-)	AWTTCAAA	S000037
ERELEE4	site	751	(-)	AWTTCAAA	S000037
EVENINGAT	site	351	(-)	AAAATATCT	S000385
GARE1OSREP1	site	342	(-)	TAACAGA	S000419
GAREAT	site	836	(+)	TAACAAR	S000439
GAREAT	site	2112	(+)	TAACAAR	S000439
GAREAT	site	144	(-)	TAACAAR	S000439
GATABOX	site	352	(+)	GATA	S000039
GATABOX	site	1754	(+)	GATA	S000039
GATABOX	site	1925	(+)	GATA	S000039
GATABOX	site	114	(-)	GATA	S000039
GATABOX	site	577	(-)	GATA	S000039
GATABOX	site	781	(-)	GATA	S000039
GATABOX	site	812	(-)	GATA	S000039
GATABOX	site	1276	(-)	GATA	S000039
GATABOX	site	1438	(-)	GATA	S000039
GATABOX	site	1523	(-)	GATA	S000039
GATABOX	site	1743	(-)	GATA	S000039
GATABOX	site	2161	(-)	GATA	S000039
GT1CONSENSUS	site	528	(+)	GRWAAW	S000198
GT1CONSENSUS	site	616	(+)	GRWAAW	S000198
GT1CONSENSUS	site	631	(+)	GRWAAW	S000198

GT1CONSENSUS	site	664	(+)	GRWAAW	S000198
GT1CONSENSUS	site	695	(+)	GRWAAW	S000198
GT1CONSENSUS	site	1003	(+)	GRWAAW	S000198
GT1CONSENSUS	site	1004	(+)	GRWAAW	S000198
GT1CONSENSUS	site	1141	(+)	GRWAAW	S000198
GT1CONSENSUS	site	1270	(+)	GRWAAW	S000198
GT1CONSENSUS	site	1538	(+)	GRWAAW	S000198
GT1CONSENSUS	site	1636	(+)	GRWAAW	S000198
GT1CONSENSUS	site	1645	(+)	GRWAAW	S000198
GT1CONSENSUS	site	1925	(+)	GRWAAW	S000198
GT1CONSENSUS	site	112	(-)	GRWAAW	S000198
GT1CONSENSUS	site	252	(-)	GRWAAW	S000198
GT1CONSENSUS	site	810	(-)	GRWAAW	S000198
GT1CONSENSUS	site	1042	(-)	GRWAAW	S000198
GT1CONSENSUS	site	1244	(-)	GRWAAW	S000198
GT1CONSENSUS	site	1468	(-)	GRWAAW	S000198
GT1CONSENSUS	site	1809	(-)	GRWAAW	S000198
GT1CONSENSUS	site	280	(-)	GRWAAW	S000198
GT1CONSENSUS	site	399	(-)	GRWAAW	S000198
GT1CONSENSUS	site	400	(-)	GRWAAW	S000198
GT1CONSENSUS	site	1436	(-)	GRWAAW	S000198
GT1CONSENSUS	site	2159	(-)	GRWAAW	S000198
GT1CORE	site	11	(+)	GGTTAA	S000125
GT1CORE	site	1191	(+)	GGTTAA	S000125
GT1CORE	site	1568	(+)	GGTTAA	S000125
GT1GMSCAM4	site	616	(+)	GAAAAA	S000453
GT1GMSCAM4	site	631	(+)	GAAAAA	S000453
GT1GMSCAM4	site	664	(+)	GAAAAA	S000453
GT1GMSCAM4	site	1004	(+)	GAAAAA	S000453
GT1GMSCAM4	site	1270	(+)	GAAAAA	S000453
GT1GMSCAM4	site	1645	(+)	GAAAAA	S000453
GT1GMSCAM4	site	280	(-)	GAAAAA	S000453
GT1GMSCAM4	site	399	(-)	GAAAAA	S000453
GTGANTG10	site	194	(+)	GTGA	S000378
GTGANTG10	site	640	(+)	GTGA	S000378
GTGANTG10	site	1213	(+)	GTGA	S000378
GTGANTG10	site	1964	(-)	GTGA	S000378
GTGANTG10	site	2053	(-)	GTGA	S000378
GTGANTG10	site	2076	(-)	GTGA	S000378
GTGANTG10	site	2145	(-)	GTGA	S000378
GTGANTG10	site	2163	(-)	GTGA	S000378
IBOX	site	779	(-)	GATAAG	S000124
IBOXCORE	site	1925	(+)	GATAA	S000199
IBOXCORE	site	113	(-)	GATAA	S000199
IBOXCORE	site	780	(-)	GATAA	S000199
IBOXCORE	site	811	(-)	GATAA	S000199
IBOXCORE	site	1437	(-)	GATAA	S000199
IBOXCORE	site	2160	(-)	GATAA	S000199
IBOXCORENT	site	778	(-)	GATAAGR	S000424
INRNTPSADB	site	761	(+)	YTCANTYY	S000395
INRNTPSADB	site	1852	(+)	YTCANTYY	S000395
INRNTPSADB	site	2082	(+)	YTCANTYY	S000395
INRNTPSADB	site	414	(-)	YTCANTYY	S000395
INRNTPSADB	site	936	(-)	YTCANTYY	S000395
INRNTPSADB	site	1007	(-)	YTCANTYY	S000395
INRNTPSADB	site	1301	(-)	YTCANTYY	S000395
INRNTPSADB	site	1287	(-)	YTCANTYY	S000395
LECPLEACS2	site	390	(+)	TAAAATAT	S000465
LECPLEACS2	site	1102	(-)	TAAAATAT	S000465
LECPLEACS2	site	1432	(-)	TAAAATAT	S000465
MARABOX1	site	792	(-)	AATAAAYAAA	S000063
MARARS	site	875	(-)	WTTTATRTTTW	S000064
MARARS	site	964	(-)	WTTTATRTTTW	S000064
MARTBOX	site	357	(+)	TTWTWTTWTT	S000067
MARTBOX	site	1777	(+)	TTWTWTTWTT	S000067
MARTBOX	site	874	(-)	TTWTWTTWTT	S000067
MARTBOX	site	929	(-)	TTWTWTTWTT	S000067
MARTBOX	site	963	(-)	TTWTWTTWTT	S000067
MYB1AT	site	1411	(+)	WAACCA	S000408
MYB1AT	site	2189	(+)	WAACCA	S000408
MYB1AT	site	10	(-)	WAACCA	S000408
MYB1AT	site	850	(-)	WAACCA	S000408
MYB1AT	site	1567	(-)	WAACCA	S000408
MYB1LEPR	site	2109	(-)	GTTAGTT	S000443
MYBCORE	site	343	(+)	CNGTTR	S000176
MYBCORE	site	1190	(+)	CNGTTR	S000176
MYBCORE	site	1614	(+)	CNGTTR	S000176

MYBGAHV	site	836	(+)	TAACAAA	S000181
MYBGAHV	site	144	(-)	TAACAAA	S000181
MYBPLANT	site	2190	(+)	MACCWAMC	S000167
MYBPLANT	site	1280	(-)	MACCWAMC	S000167
MYBPZM	site	2182	(+)	CCWACC	S000179
MYBST1	site	1438	(-)	GGATA	S000180
MYCCONSENSUSAT	site	817	(+)	CANNTG	S000407
MYCCONSENSUSAT	site	846	(+)	CANNTG	S000407
MYCCONSENSUSAT	site	1152	(+)	CANNTG	S000407
MYCCONSENSUSAT	site	1389	(+)	CANNTG	S000407
MYCCONSENSUSAT	site	1911	(+)	CANNTG	S000407
MYCCONSENSUSAT	site	2115	(+)	CANNTG	S000407
MYCCONSENSUSAT	site	817	(-)	CANNTG	S000407
MYCCONSENSUSAT	site	846	(-)	CANNTG	S000407
MYCCONSENSUSAT	site	1152	(-)	CANNTG	S000407
MYCCONSENSUSAT	site	1389	(-)	CANNTG	S000407
MYCCONSENSUSAT	site	1911	(-)	CANNTG	S000407
MYCCONSENSUSAT	site	2115	(-)	CANNTG	S000407
NAPINMOTIFBN	site	226	(+)	TACACAT	S000070
NAPINMOTIFBN	site	239	(-)	TACACAT	S000070
NAPINMOTIFBN	site	435	(-)	TACACAT	S000070
NODCON1GM	site	584	(+)	AAAGAT	S000461
NODCON1GM	site	1751	(+)	AAAGAT	S000461
NODCON1GM	site	578	(-)	AAAGAT	S000461
NODCON1GM	site	746	(-)	AAAGAT	S000461
NODCON1GM	site	1029	(-)	AAAGAT	S000461
NODCON2GM	site	1	(+)	CTCTT	S000462
NODCON2GM	site	466	(+)	CTCTT	S000462
NODCON2GM	site	474	(+)	CTCTT	S000462
NODCON2GM	site	2151	(+)	CTCTT	S000462
NODCON2GM	site	41	(-)	CTCTT	S000462
NTBBF1ARROLB	site	2032	(+)	ACTTTA	S000273
NTBBF1ARROLB	site	563	(-)	ACTTTA	S000273
OSE1ROOTNODULE	site	584	(+)	AAAGAT	S000467
OSE1ROOTNODULE	site	1751	(+)	AAAGAT	S000467
OSE1ROOTNODULE	site	578	(-)	AAAGAT	S000467
OSE1ROOTNODULE	site	746	(-)	AAAGAT	S000467
OSE1ROOTNODULE	site	1029	(-)	AAAGAT	S000467
OSE2ROOTNODULE	site	1	(+)	CTCTT	S000468
OSE2ROOTNODULE	site	466	(+)	CTCTT	S000468
OSE2ROOTNODULE	site	474	(+)	CTCTT	S000468
OSE2ROOTNODULE	site	2151	(+)	CTCTT	S000468
OSE2ROOTNODULE	site	41	(-)	CTCTT	S000468
POLASIG1	site	328	(+)	AATAAA	S000080
POLASIG1	site	668	(+)	AATAAA	S000080
POLASIG1	site	913	(+)	AATAAA	S000080
POLASIG1	site	932	(+)	AATAAA	S000080
POLASIG1	site	1297	(+)	AATAAA	S000080
POLASIG1	site	1764	(+)	AATAAA	S000080
POLASIG1	site	1788	(+)	AATAAA	S000080
POLASIG1	site	33	(-)	AATAAA	S000080
POLASIG1	site	51	(-)	AATAAA	S000080
POLASIG1	site	358	(-)	AATAAA	S000080
POLASIG1	site	599	(-)	AATAAA	S000080
POLASIG1	site	792	(-)	AATAAA	S000080
POLASIG1	site	796	(-)	AATAAA	S000080
POLASIG1	site	988	(-)	AATAAA	S000080
POLASIG1	site	1062	(-)	AATAAA	S000080
POLASIG1	site	1175	(-)	AATAAA	S000080
POLASIG1	site	1444	(-)	AATAAA	S000080
POLASIG1	site	1465	(-)	AATAAA	S000080
POLASIG1	site	1731	(-)	AATAAA	S000080
POLASIG1	site	1816	(-)	AATAAA	S000080
POLASIG2	site	134	(+)	AATTAAA	S000081
POLASIG2	site	643	(+)	AATTAAA	S000081
POLASIG2	site	1052	(+)	AATTAAA	S000081
POLASIG2	site	1109	(+)	AATTAAA	S000081
POLASIG2	site	1695	(+)	AATTAAA	S000081
POLASIG2	site	2174	(+)	AATTAAA	S000081
POLASIG2	site	1106	(-)	AATTAAA	S000081
POLASIG2	site	1988	(-)	AATTAAA	S000081
POLASIG2	site	2171	(-)	AATTAAA	S000081
POLASIG3	site	15	(+)	AATAAT	S000088
POLASIG3	site	721	(+)	AATAAT	S000088
POLASIG3	site	1095	(+)	AATAAT	S000088
POLASIG3	site	1098	(+)	AATAAT	S000088
POLASIG3	site	1146	(+)	AATAAT	S000088

POLASIG3	site	1217	(+)	AATAAT	S000088
POLASIG3	site	1417	(+)	AATAAT	S000088
POLASIG3	site	100	(-)	AATAAT	S000088
POLASIG3	site	140	(-)	AATAAT	S000088
POLASIG3	site	233	(-)	AATAAT	S000088
POLASIG3	site	287	(-)	AATAAT	S000088
POLASIG3	site	361	(-)	AATAAT	S000088
POLASIG3	site	429	(-)	AATAAT	S000088
POLASIG3	site	756	(-)	AATAAT	S000088
POLASIG3	site	1039	(-)	AATAAT	S000088
POLASIG3	site	1757	(-)	AATAAT	S000088
POLASIG3	site	1781	(-)	AATAAT	S000088
POLASIG3	site	1972	(-)	AATAAT	S000088
POLASIG3	site	1997	(-)	AATAAT	S000088
POLLEN1LELAT52	site	694	(+)	AGAAA	S000245
POLLEN1LELAT52	site	1227	(+)	AGAAA	S000245
POLLEN1LELAT52	site	1537	(+)	AGAAA	S000245
POLLEN1LELAT52	site	1559	(+)	AGAAA	S000245
POLLEN1LELAT52	site	1591	(+)	AGAAA	S000245
POLLEN1LELAT52	site	1630	(+)	AGAAA	S000245
POLLEN1LELAT52	site	1644	(+)	AGAAA	S000245
POLLEN1LELAT52	site	1749	(+)	AGAAA	S000245
POLLEN1LELAT52	site	1798	(+)	AGAAA	S000245
POLLEN1LELAT52	site	160	(-)	AGAAA	S000245
POLLEN1LELAT52	site	282	(-)	AGAAA	S000245
POLLEN1LELAT52	site	340	(-)	AGAAA	S000245
POLLEN1LELAT52	site	463	(-)	AGAAA	S000245
POLLEN1LELAT52	site	1246	(-)	AGAAA	S000245
POLLEN1LELAT52	site	1470	(-)	AGAAA	S000245
POLLEN1LELAT52	site	1667	(-)	AGAAA	S000245
PREATPRODH	site	1929	(-)	ACTCAT	S000450
RAV1AAT	site	1386	(+)	CAACA	S000314
RAV1AAT	site	1904	(+)	CAACA	S000314
RAV1AAT	site	2018	(+)	CAACA	S000314
RBCSCONSENSUS	site	542	(-)	AATCCAA	S000127
REALPHALGLHCB21	site	1412	(+)	AACCAA	S000362
REALPHALGLHCB21	site	2190	(+)	AACCAA	S000362
REALPHALGLHCB21	site	200	(-)	AACCAA	S000362
ROOTMOTIFTAPOX1	site	94	(+)	ATATT	S000098
ROOTMOTIFTAPOX1	site	166	(+)	ATATT	S000098
ROOTMOTIFTAPOX1	site	187	(+)	ATATT	S000098
ROOTMOTIFTAPOX1	site	231	(+)	ATATT	S000098
ROOTMOTIFTAPOX1	site	353	(+)	ATATT	S000098
ROOTMOTIFTAPOX1	site	383	(+)	ATATT	S000098
ROOTMOTIFTAPOX1	site	447	(+)	ATATT	S000098
ROOTMOTIFTAPOX1	site	509	(+)	ATATT	S000098
ROOTMOTIFTAPOX1	site	657	(+)	ATATT	S000098
ROOTMOTIFTAPOX1	site	808	(+)	ATATT	S000098
ROOTMOTIFTAPOX1	site	897	(+)	ATATT	S000098
ROOTMOTIFTAPOX1	site	921	(+)	ATATT	S000098
ROOTMOTIFTAPOX1	site	1102	(+)	ATATT	S000098
ROOTMOTIFTAPOX1	site	1374	(+)	ATATT	S000098
ROOTMOTIFTAPOX1	site	1425	(+)	ATATT	S000098
ROOTMOTIFTAPOX1	site	1432	(+)	ATATT	S000098
ROOTMOTIFTAPOX1	site	1599	(+)	ATATT	S000098
ROOTMOTIFTAPOX1	site	1620	(+)	ATATT	S000098
ROOTMOTIFTAPOX1	site	1664	(+)	ATATT	S000098
ROOTMOTIFTAPOX1	site	1685	(+)	ATATT	S000098
ROOTMOTIFTAPOX1	site	1703	(+)	ATATT	S000098
ROOTMOTIFTAPOX1	site	1726	(+)	ATATT	S000098
ROOTMOTIFTAPOX1	site	1755	(+)	ATATT	S000098
ROOTMOTIFTAPOX1	site	1779	(+)	ATATT	S000098
ROOTMOTIFTAPOX1	site	93	(-)	ATATT	S000098
ROOTMOTIFTAPOX1	site	150	(-)	ATATT	S000098
ROOTMOTIFTAPOX1	site	372	(-)	ATATT	S000098
ROOTMOTIFTAPOX1	site	393	(-)	ATATT	S000098
ROOTMOTIFTAPOX1	site	506	(-)	ATATT	S000098
ROOTMOTIFTAPOX1	site	573	(-)	ATATT	S000098
ROOTMOTIFTAPOX1	site	622	(-)	ATATT	S000098
ROOTMOTIFTAPOX1	site	877	(-)	ATATT	S000098
ROOTMOTIFTAPOX1	site	891	(-)	ATATT	S000098
ROOTMOTIFTAPOX1	site	896	(-)	ATATT	S000098
ROOTMOTIFTAPOX1	site	920	(-)	ATATT	S000098
ROOTMOTIFTAPOX1	site	966	(-)	ATATT	S000098
ROOTMOTIFTAPOX1	site	1101	(-)	ATATT	S000098
ROOTMOTIFTAPOX1	site	1274	(-)	ATATT	S000098
ROOTMOTIFTAPOX1	site	1373	(-)	ATATT	S000098

ROOTMOTIFTAPOX1	site	1483	(-)	ATATT	S000098
ROOTMOTIFTAPOX1	site	1598	(-)	ATATT	S000098
ROOTMOTIFTAPOX1	site	1619	(-)	ATATT	S000098
ROOTMOTIFTAPOX1	site	1663	(-)	ATATT	S000098
ROOTMOTIFTAPOX1	site	1682	(-)	ATATT	S000098
ROOTMOTIFTAPOX1	site	1700	(-)	ATATT	S000098
S1FBOXSORPS1L21	site	1139	(+)	ATGGTA	S000223
S1FBOXSORPS1L21	site	1634	(+)	ATGGTA	S000223
SEBFCONSSTPR10A	site	1673	(+)	YTGTCWC	S000391
SEF1MOTIF	site	897	(+)	ATATTTAWW	S000006
SEF1MOTIF	site	1696	(-)	ATATTTAWW	S000006
SEF1MOTIF	site	1369	(-)	ATATTTAWW	S000006
SEF3MOTIFGM	site	2179	(+)	AACCCA	S000115
SEF3MOTIFGM	site	2184	(+)	AACCCA	S000115
SEF3MOTIFGM	site	2195	(+)	AACCCA	S000115
SEF4MOTIFGM7S	site	273	(+)	RTTTTTR	S000103
SEF4MOTIFGM7S	site	290	(+)	RTTTTTR	S000103
SEF4MOTIFGM7S	site	355	(+)	RTTTTTR	S000103
SEF4MOTIFGM7S	site	596	(+)	RTTTTTR	S000103
SEF4MOTIFGM7S	site	905	(+)	RTTTTTR	S000103
SEF4MOTIFGM7S	site	1172	(+)	RTTTTTR	S000103
SEF4MOTIFGM7S	site	1728	(+)	RTTTTTR	S000103
SEF4MOTIFGM7S	site	982	(-)	RTTTTTR	S000103
SEF4MOTIFGM7S	site	934	(-)	RTTTTTR	S000103
SEF4MOTIFGM7S	site	962	(-)	RTTTTTR	S000103
SEF4MOTIFGM7S	site	976	(-)	RTTTTTR	S000103
SEF4MOTIFGM7S	site	1514	(-)	RTTTTTR	S000103
SEF4MOTIFGM7S	site	1691	(-)	RTTTTTR	S000103
SITEIIATCYTC	site	1868	(+)	TGGGCY	S000474
SITEIIATCYTC	site	1842	(-)	TGGGCY	S000474
SORLIP1AT	site	2040	(-)	GCCAC	S000482
SORLREP3AT	site	442	(+)	TGTATATAT	S000488
SP8BFIBSP8BIB	site	1738	(-)	TACTATT	S000184
SREATMSD	site	1437	(+)	TTATCC	S000470
SURE1STPAT21	site	281	(-)	AATAGAAAA	S000186
SURECOREATSULTR11	site	1454	(-)	GAGAC	S000499
SURECOREATSULTR11	site	1675	(-)	GAGAC	S000499
SURECOREATSULTR11	site	1916	(-)	GAGAC	S000499
T/GBOXATPIN2	site	2037	(+)	AACGTG	S000458
TAAAGSTKST1	site	563	(+)	TAAAG	S000387
TAAAGSTKST1	site	583	(+)	TAAAG	S000387
TAAAGSTKST1	site	1571	(+)	TAAAG	S000387
TAAAGSTKST1	site	1627	(+)	TAAAG	S000387
TAAAGSTKST1	site	206	(-)	TAAAG	S000387
TAAAGSTKST1	site	257	(-)	TAAAG	S000387
TAAAGSTKST1	site	476	(-)	TAAAG	S000387
TAAAGSTKST1	site	580	(-)	TAAAG	S000387
TAAAGSTKST1	site	1061	(-)	TAAAG	S000387
TAAAGSTKST1	site	1443	(-)	TAAAG	S000387
TAAAGSTKST1	site	1987	(-)	TAAAG	S000387
TAAAGSTKST1	site	2033	(-)	TAAAG	S000387
TAAAGSTKST1	site	2158	(-)	TAAAG	S000387
TAAAGSTKST1	site	2170	(-)	TAAAG	S000387
TATABOX2	site	301	(+)	TATAAAT	S000109
TATABOX2	site	879	(+)	TATAAAT	S000109
TATABOX2	site	1369	(+)	TATAAAT	S000109
TATABOX2	site	298	(-)	TATAAAT	S000109
TATABOX2	site	1421	(-)	TATAAAT	S000109
TATABOX3	site	95	(+)	TATTAAT	S000110
TATABOX3	site	1974	(+)	TATTAAT	S000110
TATABOX3	site	1660	(-)	TATTAAT	S000110
TATABOX4	site	119	(+)	TATATAA	S000111
TATABOX4	site	828	(+)	TATATAA	S000111
TATABOX4	site	1198	(+)	TATATAA	S000111
TATABOX4	site	2058	(+)	TATATAA	S000111
TATABOX4	site	735	(-)	TATATAA	S000111
TATABOX4	site	1116	(-)	TATATAA	S000111
TATABOX4	site	1197	(-)	TATATAA	S000111
TATABOX5	site	101	(+)	TTATTT	S000203
TATABOX5	site	141	(+)	TTATTT	S000203
TATABOX5	site	288	(+)	TTATTT	S000203
TATABOX5	site	362	(+)	TTATTT	S000203
TATABOX5	site	600	(+)	TTATTT	S000203
TATABOX5	site	609	(+)	TTATTT	S000203
TATABOX5	site	704	(+)	TTATTT	S000203
TATABOX5	site	757	(+)	TTATTT	S000203
TATABOX5	site	793	(+)	TTATTT	S000203

TATABOX5	site	797	(+)	TTATTT	S000203
TATABOX5	site	989	(+)	TTATTT	S000203
TATABOX5	site	1040	(+)	TTATTT	S000203
TATABOX5	site	1063	(+)	TTATTT	S000203
TATABOX5	site	1176	(+)	TTATTT	S000203
TATABOX5	site	1466	(+)	TTATTT	S000203
TATABOX5	site	1732	(+)	TTATTT	S000203
TATABOX5	site	667	(-)	TTATTT	S000203
TATABOX5	site	697	(-)	TTATTT	S000203
TATABOX5	site	931	(-)	TTATTT	S000203
TATABOX5	site	1094	(-)	TTATTT	S000203
TATABOX5	site	1145	(-)	TTATTT	S000203
TATABOX5	site	1216	(-)	TTATTT	S000203
TATABOX5	site	1416	(-)	TTATTT	S000203
TATABOX5	site	1649	(-)	TTATTT	S000203
TATABOX5	site	1787	(-)	TTATTT	S000203
TATABOXOSPAL	site	798	(+)	TATTTAA	S000400
TATABOXOSPAL	site	898	(+)	TATTTAA	S000400
TATABOXOSPAL	site	1733	(+)	TATTTAA	S000400
TATABOXOSPAL	site	1092	(-)	TATTTAA	S000400
TATABOXOSPAL	site	1553	(-)	TATTTAA	S000400
TATABOXOSPAL	site	1697	(-)	TATTTAA	S000400
TATABOXOSPAL	site	1785	(-)	TATTTAA	S000400
TATAPVTRNALEU	site	734	(+)	TTTATATA	S000340
WBOXATNPR1	site	170	(+)	TTGAC	S000390
WBOXATNPR1	site	335	(+)	TTGAC	S000390
WBOXATNPR1	site	366	(+)	TTGAC	S000390
WBOXHVISO1	site	336	(+)	TGACT	S000442
WBOXHVISO1	site	626	(+)	TGACT	S000442
WBOXNTERF3	site	336	(+)	TGACY	S000457
WBOXNTERF3	site	626	(+)	TGACY	S000457
WRKY71OS	site	24	(+)	TGAC	S000447
WRKY71OS	site	171	(+)	TGAC	S000447
WRKY71OS	site	336	(+)	TGAC	S000447
WRKY71OS	site	367	(+)	TGAC	S000447
WRKY71OS	site	424	(+)	TGAC	S000447
WRKY71OS	site	626	(+)	TGAC	S000447
WRKY71OS	site	1222	(+)	TGAC	S000447
WRKY71OS	site	2144	(-)	TGAC	S000447
